# Responses of Soil Microbial Community to Rocky Desertification Succession and Their Relationships With Plant Functional Diversity in Southwest China Karst Ecosystem

**DOI:** 10.1002/ece3.73013

**Published:** 2026-01-30

**Authors:** Dandan Zhu, Maoyin Sheng, Yaoyao Liu, Linjiao Wang

**Affiliations:** ^1^ Institute of Karst Research Guizhou Normal University Guiyang China; ^2^ School of Environmental Science and Engineering Hefei Institute of Technology Hefei China

**Keywords:** functional diversity, functional group, karst, rocky desertification, soil microorganism

## Abstract

Soil microorganisms play a critical regulatory role in various ecosystem functions, making them essential for maintaining ecosystem stability. Plant functional diversity influences soil physicochemical properties and shapes the structure and function of soil microbial communities. However, the response patterns of soil microorganisms to karst rocky desertification (KRD) succession remain poorly understood, particularly the relationship between microbial community dynamics and aboveground plant functional diversity. Here, the present study focuses on four typical succession KRD stages in a representative karst area of southwest China. By analyzing the diversity and community composition of soil microorganisms (bacteria and fungi), measuring soil physicochemical properties, and assessing aboveground plant functional diversity, the study aims to clarify the response patterns of soil microbial communities to KRD and their relationships with plant functional diversity in the KRD ecosystem of southwest China. The following main results and conclusions were obtained: (1) The composition of soil microbial communities and diversity varied significantly across different rocky desertification degrees. Functional diversity of aboveground plants also exhibited significant differences across varying rocky desertification degrees. (2) Both taxonomic composition and diversity indices of soil microorganisms (bacteria and fungi) showed significant (*p* < 0.05) or highly significant (*p* < 0.01) correlations with soil physicochemical properties and plant functional diversity across the four rocky desertification degrees. (3) During the karst rocky desertification succession, soil bacterial diversity was directly influenced by plant functional diversity and soil physicochemical properties. While, soil fungal diversity exhibited direct regulation by soil physicochemical properties and indirect effects from plant functional diversity mediated through soil properties. During the rocky desertification succession process, there are very complex interactions present among plants, soils, and microorganisms. Elucidating these relationships is of great significance for vegetation restoration and rocky desertification control in karst regions.

## Graphical abstract

During the succession of karst rocky desertification, soil bacterial diversity was directly influenced by both plant functional diversity and soil physicochemical properties. In contrast, soil fungal diversity was directly regulated by soil physicochemical properties and indirectly affected by plant functional diversity via the mediation of soil properties.

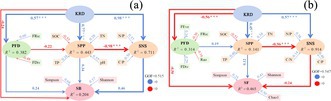

## 1 Introduction

Soil microorganisms play a critical regulatory role in various ecosystem functions, participating in organic matter decomposition, nutrient cycling, and maintaining soil fertility, which are of great significance for sustaining ecosystem stability (van der Heijden et al. 2008; Labouyrie et al. 2023). Soil microorganisms, primarily including bacteria and fungi, play a key role in regulating soil nutrients and promoting plant growth (Kirk et al. 2004; Yang et al. 2021). Their community structure and function are highly sensitive to environmental changes and are often used as indicative indicators for assessing ecosystem homeostasis (Schloter et al. 2003; Xiao et al. 2025). With advances in molecular biology techniques, high‐throughput sequencing has enabled deeper investigations into soil microbial diversity and ecological functions (Prosser 2015). The community structure of soil microorganisms undergoes significant changes under different environmental conditions, with land‐use types, soil physicochemical properties, and external disturbances all significantly influencing microbial diversity and function (Fanin et al. 2017; Bei et al. 2018). In karst environments, the unique soil conditions and rocky desertification succession processes result in microbial communities exhibiting distinct ecological characteristics (Tang et al. 2019). Studies have shown that in the karst ecosystems, plant diversity and soil properties significantly influence soil microorganisms (Zheng et al. 2024). In recent years, increasing attention has been paid to the relationship between rocky desertification succession and external environmental and anthropogenic factors from the perspective of soil microorganisms. However, studies on the specific response mechanisms between soil physicochemical characteristics and microbial community across different stages of rocky desertification succession remain insufficient.

Karst rocky desertification (KRD) is the primary form of land degradation in southwest China karst regions, characterized by reduced vegetation cover, severe soil erosion, and expanding rocky exposure, leading to ecosystem deterioration and declining soil productivity (Wang et al. 2004; Huang and Cai 2006). KRD results from the combined effects of various natural and anthropogenic factors, with human disturbances such as over‐cultivation, overgrazing, and deforestation being the main drivers (Jiang et al. 2014). The southwest China karst region is the largest contiguous karst area in the world. Intensive human activities in this region have caused severe rocky desertification. KRD is not only a critical ecological and environmental issue but also poses a significant threat to regional socio‐economic development and sustainability, which has led to a significant decline in ecosystem service functions (Huang and Cai 2007). For example, a quantitative assessment in a typical KRD area of northwestern Guangxi showed that the total value of ecosystem services decreased from 109.652 billion yuan in 1985 to 88.789 billion yuan in 1990, and even though it recovered slightly thereafter, it still did not return to the level of the 1980s by 2005 (Zhang et al. 2009). Soil restoration and vegetation rehabilitation in KRD areas are now urgent priorities. Scientific studies on KRD have become a major focus in karst ecology, with extensive studies conducted on soil nutrient cycling, plant diversity, and microbial diversity (Sheng et al. 2018; Zheng et al. 2024). Sheng et al. (2015, 2018) found that soil physicochemical properties vary significantly across different KRD stages, showing an initial degradation followed by gradual improvement. Despite these advances, most existing studies have focused on macroscopic correlations between plant community diversity (e.g., species richness, community composition) and microbial diversity, or isolated effects of individual soil physicochemical factors on microbial communities. The fine‐scale response mechanisms of soil microbial communities (especially the differential responses of bacteria and fungi) to the continuous changes in plant functional traits during KRD succession remain insufficiently explored. Understanding these patterns and mechanisms is crucial for scientifically and effectively implementing karst vegetation restoration and KRD control measures.

Plant functional diversity, defined as the variation in functional traits among species within a plant community (Zhang et al. 2012), serves as a predictor of ecosystem functioning (Lei et al. 2016). It plays a crucial role in maintaining ecosystem functions, enhancing stability, and improving resilience (Díaz and Cabido 2001; Lavorel et al. 2011). Plant functional diversity influences soil physical and chemical properties, as well as the structure and function of soil microbial communities (Xue et al. 2017; Cheng et al. 2022). Different plant traits, such as root architecture, photosynthetic efficiency, and nutrient absorption capacity, significantly shape the diversity of soil microbial communities (Wagg et al. 2014). Plant diversity and functional traits indirectly affect microbial composition and activity by altering soil physicochemical properties (Delgado‐Baquerizo et al. 2016; Sasse et al. 2018). Through photosynthesis, plants fix carbon dioxide and transfer organic matter into the soil, serving as a key carbon and energy source for soil microorganisms (Gougoulias et al. 2014; Yang and Zhu 2022). In turn, soil microbes decompose organic matter, mineralize nutrients (e.g., nitrogen and phosphorus), and enhance plant growth, thereby influencing plant functional diversity (Richardson et al. 2009). Thus, plants, soil, and microorganisms form an interdependent ecosystem (Bei et al. 2018; Yan et al. 2020; Zheng et al. 2024). Plants supply carbon and energy to soil microbes via root exudates, while microbes provide essential nutrients through organic matter decomposition and nitrogen fixation, promoting plant growth and diversity (Zhao et al. 2020). Therefore, we propose the following hypotheses: (1) During KRD succession, soil bacterial and fungal communities exhibit significant shifts in composition and diversity indices. (2) Changes in soil microbial communities are significantly correlated with aboveground plant functional diversity across KRD succession stages.

To test these two hypotheses, in the present study, a typical KRD region in southwest China was selected as the study area. The unique focus of this study lies in linking plant functional diversity (instead of macro species diversity) to soil microbial community dynamics across continuous KRD succession stages, and quantifying the mediating role of soil physicochemical properties in this relationship. The study objectives were to: (1) Examine the variation patterns of soil microbial community structure and diversity across different KRD succession stages; (2) Analyze the correlation between soil microbial communities and aboveground plant functional diversity during KRD succession; (3) Elucidate the response mechanisms of soil microorganisms to KRD succession and their driving factors. The findings aim to provide a theoretical foundation for vegetation restoration and rocky desertification control in southwest China karst regions.

## 2 Materials and Methods

### 2.1 Study Area

**FIGURE 1:**
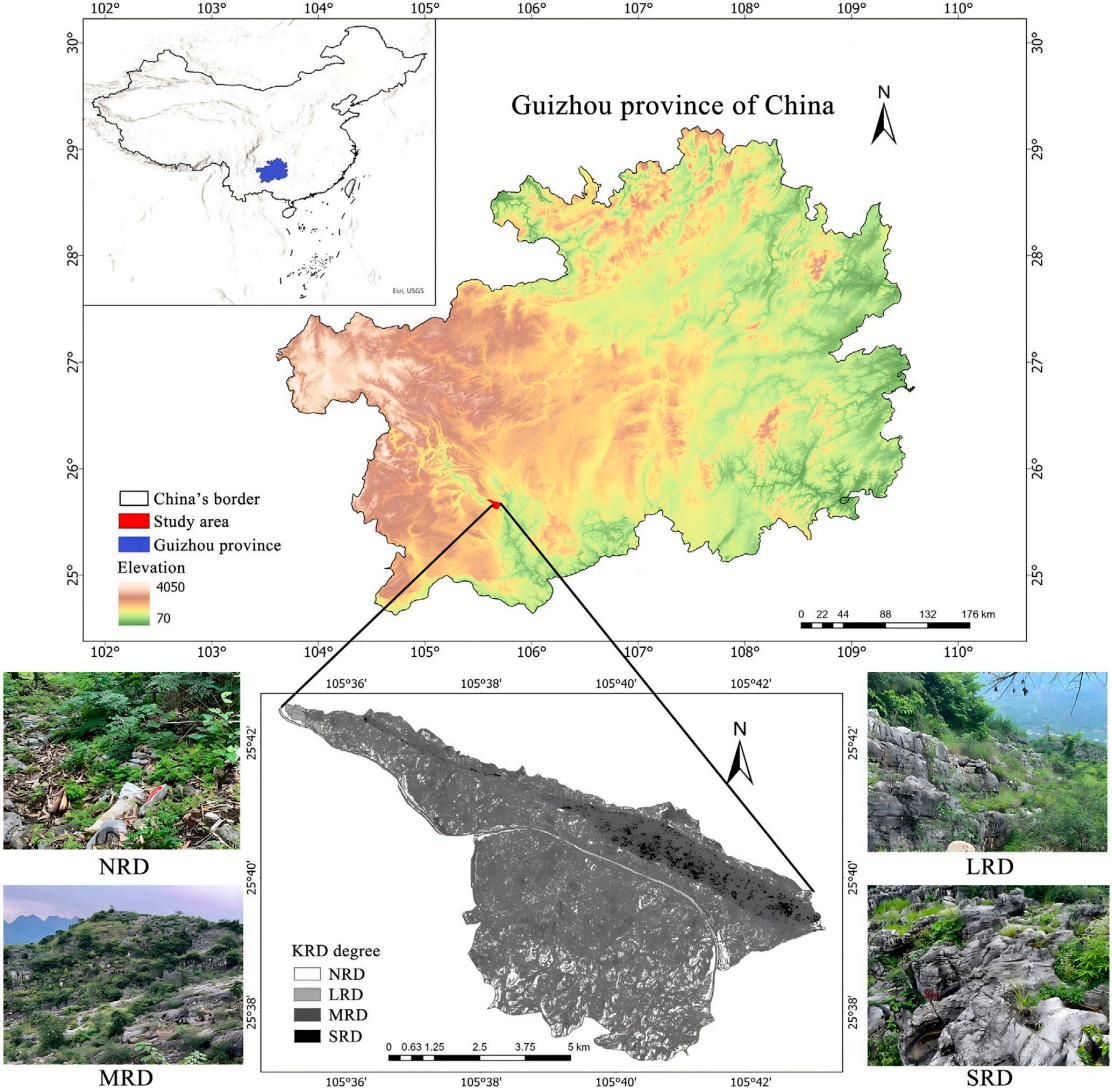
Location of study area and landscapes of typical KRD stages in the study area. KRD, karst rocky desertification; LRD, light rocky desertification; MRD, moderate rocky desertification; NRD, nil rocky desertification; SRD, severe rocky desertification.

The study area is located in Beipanjiang Town, Zhenfeng County, Guizhou Province, China (105°38′53″ E, 25°39′37″ N), characterized by a typical karst plateau‐canyon landform (Figure 1). It covers a total area of 51.62 km^2^, with karst landscapes accounting for 87.92% of the total area. The elevation ranges from 450 to 1450 m, with a relative height difference of 1000 m. The area experiences warm and dry winters and springs, while summers and autumns are hot and humid, with abundant thermal resources. The annual average temperature is 18.4°C, with the highest temperature reaching 32.4°C and the lowest dropping to 6.6°C. The average annual precipitation is approximately 1100 mm, with 83% of the rainfall concentrated between May and October. The dominant rock formations in the area consist of Triassic dolomite, argillaceous dolomite, and shale. The primary soil types include yellow soil and yellow calcareous soil, while the vegetation is classified as subtropical evergreen‐deciduous coniferous and broad‐leaved mixed forest.

Due to intense human activities, the area suffers from severe KRD. KRD has significantly altered the aboveground vegetation types and species composition. In light KRD stages, the vegetation is dominated by secondary forests, shrublands, and herbaceous plants. The main arbor species include *Cupressus funebris* , *Toona sinensis*, and *Catalpa fargesii* . The dominant shrubs are *Pyracantha fortuneana* , *Rosa cymosa*, and *Coriaria nepalensis*. The representative herbaceous plants consist of *Imperata cylindrica* , *Setaria viridis* , and ferns. In moderate KRD stages, shrub‐grasslands and sparse arbors prevail. The major shrubs include *Opuntia dillenii* , *Rosa laevigata* , and *Rubus* spp. The dominant herbaceous plants are *Heteropogon contortus* , *Themeda triandra* , and *Bidens pilosa* . In severe KRD stages, sparse herbaceous plants and drought‐tolerant shrubs are the main vegetation. The primary shrubs include *Opuntia dillenii* and *Rosa cymosa*. The dominant herbaceous plants include *Cynodon dactylon* , *Carex* spp., lichens, and bryophytes.

### 2.2 Sampling Plot Establishment and Soil Sample Collection

Targeting the four typical stages of KRD succession: NRD (nil rocky desertification), LRD (light rocky desertification), MRD (moderate rocky desertification), and SRD (severe rocky desertification) (classification criteria refer to Sheng et al. (2015)), we established three replicated plots (20 m × 20 m each) for each stage in the study area (Table S1). Soil samples were collected from each plot using the five‐point sampling method at a depth of 0–15 cm. After removing surface litter, moss, and debris such as stones, the soil from the five sampling points was thoroughly mixed and evenly divided into two subsamples, which were transported to the laboratory in insulated containers. One subsample was air‐dried, sieved, and sealed for the analysis of soil physicochemical properties. The other subsample was sieved through a 10‐mesh sieve, placed into cryotubes for biological analysis, treated with liquid nitrogen, and stored at −80°C for soil microbial analysis. Additionally, undisturbed soil cores were collected from each plot using a cutting ring for soil bulk density determination (Sheng et al. 2015).

### 2.3 Determination of Soil Physicochemical Properties

Soil organic carbon (SOC) was determined using the potassium dichromate oxidation‐external heating method. Soil total nitrogen (TN) was measured by the Kjeldahl digestion method (using potassium sulfate, copper sulfate, and selenium powder as catalysts) followed by automatic analysis with a nitrogen analyzer. Soil total phosphorus (TP) was determined using sulfuric acid‐perchloric acid digestion followed by molybdenum‐antimony anti‐colorimetry. Soil pH was measured at a water‐to‐soil ratio of 2.5:1 using the electrode potentiometry method. Soil bulk density (BD) was determined by the cutting‐ring method. Soil water content (SWC) was calculated as the percentage of weight loss in fresh soil after drying at 105°C for 24 h. All these methods referred to Sheng et al. (2015). Detailed measurement results are presented in Table 1 and Table S2.

**TABLE 1:** Soil physical and chemical properties in the different degrees of karst rocky desertification.

Soil properties	Karst rocky desertification degrees			
	NRD	LRD	MRD	SRD
SOC (g·kg^−1^)	34.67 ± 1.02^a^	31.43 ± 0.40^b^	36.74 ± 0.82^a^	25.56 ± 0.55^c^
TN (g·kg^−1^)	1.77 ± 0.15^a^	1.36 ± 0.07^ab^	1.46 ± 0.07^a^	1.88 ± 0.25^ac^
TP (g·kg^−1^)	1.13 ± 0.10^a^	0.76 ± 0.03^b^	0.64 ± 0.02^bc^	1.11 ± 0.22^ab^
pH	7.42 ± 0.01^a^	7.34 ± 0.02^b^	7.57 ± 0.01^c^	7.56 ± 0.03^c^
SWC (%)	24.01 ± 3.48^a^	15.34 ± 1.53^a^	21.55 ± 4.07^a^	17.96 ± 2.76^a^
BD (%)	1.06 ± 0.05^a^	1.20 ± 0.04^a^	1.11 ± 0.06^a^	1.11 ± 0.04^a^
C:N	20.64 ± 1.60^a^	23.57 ± 1.17^ab^	25.55 ± 1.28^b^	15.40 ± 1.76^c^
C:P	32.35 ± 2.49^a^	41.61 ± 1.21^b^	58.04 ± 2.61^c^	29.49 ± 4.23^ac^
N:P	1.57 ± 0.05^a^	1.78 ± 0.04^ab^	2.28 ± 0.04^c^	1.85 ± 0.12^b^

*Note:* No same letter represents the significant (*p* < 0.05) differences in the same soil property between karst rocky desertification degrees.

Abbreviations: BD, bulk density; C:N, SOC:TN; C:P, SOC:TP; LRD, light rocky desertification; MRD, moderate rocky desertification; N:P, TN:TP; NRD, nil rocky desertification; SOC, soil organic carbon content; SRD, severe rocky desertification; SWC, soil water content; TN, total nitrogen content; TP, total phosphorus content.

### 2.4 DNA Extraction, Amplification, and Sequencing of Soil Microorganisms

High‐throughput genomic sequencing was employed to analyze soil microbial diversity (Zheng et al. 2024). For bacterial community analysis, the V4 region of the 16S rRNA gene was amplified using universal primers 515F and 806R. For fungal community analysis, the ITS1 region was amplified using primers ITS5‐1737F and ITS2‐2043R. The PCR (polymerase chain reaction) reaction mixture contained 15 μL Phusion High‐Fidelity PCR Master Mix (New England Biolabs), 0.2 μM of each primer, and 10 ng of genomic DNA template. The thermal cycling conditions consisted of: initial denaturation at 98°C for 1 min, followed by 30 cycles of denaturation at 98°C for 10 s, annealing at 50°C for 30 s, and extension at 72°C for 30 s, with a final extension at 72°C for 5 min. PCR products were verified by electrophoresis on 2% agarose gels. Qualified products were purified using magnetic beads, quantified by enzyme‐linked methods, and pooled in equimolar amounts. The pooled samples were re‐checked by 2% agarose gel electrophoresis, and target bands were excised for recovery. Short‐fragment libraries were constructed according to the characteristics of the amplified regions. The constructed libraries were quantified using Qubit fluorometry and Q‐PCR. Qualified libraries were subjected to paired‐end sequencing on the Illumina NovaSeq platform. After sequencing, reads were assembled and filtered. Noise reduction was performed using DADA2, generating amplicon sequence variants (ASVs) from the de‐replicated sequences.

### 2.5 Analysis of Soil Microbial Community Structure and Diversity

The obtained ASVs (amplicon sequence variants) were taxonomically annotated and analyzed for abundance to reveal soil microbial community composition (Yang et al. 2021; Zheng et al. 2024). Species abundance and α‐diversity indices (Chao1, Shannon, and Simpson) were calculated using ASV data with the following formulas (Yang et al. 2021; Zheng et al. 2024):

$$\text{Chao}1=S_{\mathrm{obs}}+\frac{F_{1}^{2}}{2F_{2}}$$

$$H_{\text{Shannon}}=-\sum_{i=1}^{S}p_{i}\ln\left(p_{i}\right)$$

$$D_{\text{Simpson}}=\sum_{i=1}^{S}p_{i}^{2}$$

In these formulas, *S*_obs_ = number of observed ASVs, *F*_1_ = number of singletons (ASVs appearing only once), *F*_2_ = number of doubletons (ASVs appearing twice), *S* = total number of ASVs in the sample, *p*_*i*_ = relative abundance of the *i*‐th ASV (*p*_*i*_ = *n*_*i*_/*N*), *n*_*i*_ = abundance of the *i*‐th ASV, *N* = total abundance of all ASVs in the sample. The calculation results of relative abundance of soil bacterial and fungal were filled in Tables S3 and S4, respectively. The calculation of α‐diversity indices (Chao1, Shannon, and Simpson) was filled in Table S5.

Differences in soil microbial community structure (β‐diversity) between samples or groups were visualized through dimensionality reduction methods of PCoA (principal coordinates analysis), PCA (principal component analysis), and NMDS (nonmetric multidimensional scaling). The Bray–Curtis dissimilarity index was used as the distance metric for PCoA. These calculation results of β‐diversity indices of bacterial and fungal were filled in Tables S6 and S7, respectively.

### 2.6 Plant Sample Collection and Functional Trait Measurement

Within each plot, we recorded tree species, individual counts, and tree height (*H*). Three randomly placed 1 m × 1 m quadrats were established per plot to survey herbaceous species composition, abundance, and height (Fang et al. 2009). Mature, sun‐exposed leaves in good physiological condition were collected from dominant species. After transport to the laboratory, leaves were oven‐dried and ground for elemental analysis of C (carbon), N (nitrogen), P (phosphorus), and Si (silicon). Five intact leaves per plant were selected for leaf area measurements.

Leaf C content was determined by the potassium dichromate oxidation method. Leaf N content was determined by the Kjeldahl method. Leaf P content was determined by the molybdenum–antimony anti‐colorimetric method. Leaf Si content was determined by the silicon‐molybdenum blue colorimetry method. Leaf area (LA) was measured by scanning leaves with a leaf area digital scanner (WinRHIZO Pro LA2400). The leaf mass per area (LMA, g/m^2^) was calculated as: LMA = leaf dry weight (g)/leaf area (cm^2^). All these methods referred to Richardson et al. (2009). All functional trait measurement results are presented in Table S8.

### 2.7 Calculation of Plant Functional Diversity Index

The calculation of plant functional diversity indices was based on the measurement results of plant functional traits (Falster and Westoby 2003; He et al. 2010), including the functional richness index (FRic), the functional evenness index (FEve), the Rao's quadratic entropy (Rao), the functional divergence index (FDiv), and the functional dispersion index (FDis) (Dong et al. 2017). The functional richness index (FRic) is obtained by calculating the area or volume of the minimum convex hull formed in the trait space. The calculation formulas for the other indices are as follows (He et al. 2010):

$$\text{FEve}=\frac{\sum_{b=1}^{S-1}\min\left(\mathrm{PE}W_{b}\cdot \frac{1}{S-1}\right)-\frac{1}{S-1}}{1-\frac{1}{S-1}}$$

$$\mathrm{PE}W_{b}=\frac{EW_{b}}{\sum_{b=1}^{S-1}EW_{b}}$$

$$EW_{b}=\frac{d_{\mathrm{ij}}}{w_{i}+w_{j}}$$

In these formulas, *S* is the number of species, PEW_b_ is the locally weighted mean evenness, EW_b_ is the weighted mean evenness, *w*_*i*_ is the relative abundance of species *i*, *d*_*ij*_ is the Euclidean distance between species *i* and species *j*.

$$\mathrm{Rao}=\sum_{i=1}^{S}\sum_{j>1}^{S}d_{\mathrm{ij}}w_{i}w_{j}$$

In the formula, *S* is the number of species, *d*_*ij*_ is the Euclidean distance between species *i* and species *j*, and *w*_*i*_ and *w*_*j*_ are the relative abundance of species *i* and *j*, respectively.

$$FD_{\mathrm{iv}}=\frac{\sum_{i=1}^{n}\left(p_{i}\cdot d_{i}\right)}{\sum_{i=1}^{n}p_{i}\cdot \bar{d}}$$

In the formula, *p*_*i*_ is the abundance of species *i*, *d*_*i*_ is the distance of species *i* to the community‐weighted mean, $\bar{d}$ is the average distance of all species.

$$c=\left[c_{i}\right]=\frac{\sum w_{j}x_{\mathrm{ik}}}{\sum w_{j}}$$

$$FD_{\text{is}}=\frac{\sum w_{j}z_{j}}{\sum w_{j}}$$

In the formulas, *C* is the weighted centroid, *w*_*j*_ is the relative abundance of species *j*, *x*_*ik*_ is the value of trait *k* for species *i*, and *z*_*j*_ is the weighted distance of species *i* to the centroid *C*. These functional diversity indices were also presented in Table S8.

### 2.8 Data Statistical Analysis and Visualization

Data organization and preliminary calculations were performed using Microsoft Excel 2010. One‐way analysis of variance (One‐Way ANOVA) was conducted in SPSS 25.00 to examine group differences in soil physicochemical properties, soil microbial diversity, and plant functional diversity. Redundancy analysis (RDA) and Mantel tests were employed to identify key factors influencing soil microbial community composition and diversity patterns, with all analyses and visualizations performed using R language. Dimensionality reduction analyses (including PCoA, PCA, and NMDS) for comparing soil microbial community structures across different samples or groups were also implemented and visualized using R programming.

## 3 Results

### 3.1 Soil Microbial Community Structure

**FIGURE 2:**
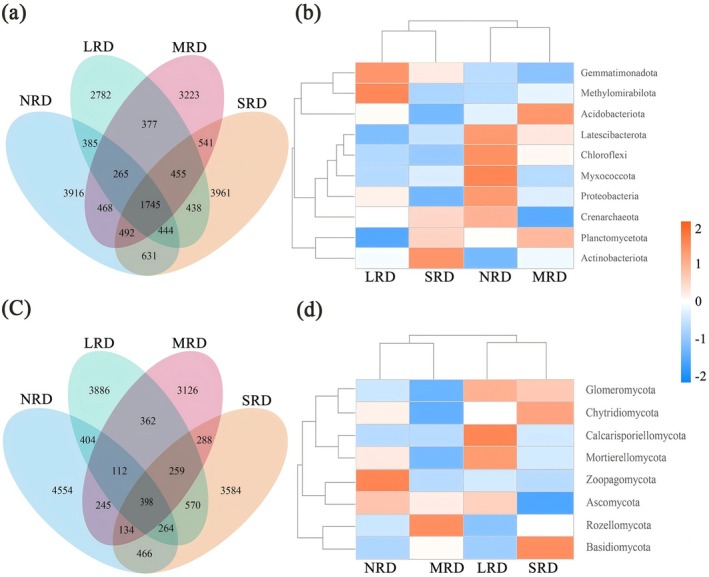
Petal Venn plot and species abundance clustering map (phylum level) based on ASVs quantity for soil bacteria (a, b) and fungi (c, d) in the different KRD stages. ASVs, amplicon sequence variants; KRD, karst rocky desertification; LRD, light rocky desertification; MRD, moderate rocky desertification; NRD, nil rocky desertification; SRD, severe rocky desertification.

In the four different stages (NRD, LRD, MRD, and SRD) of rocky desertification soils, a total of 1745 bacterial ASVs (Figure 2a) and 398 fungal ASVs (Figure 2c) were obtained. The top 10 bacterial phyla in terms of richness were Gemmatimonadota, Methylomirabilota, Acidobacterota, Latescibacterota, Chloroflexi, Myxococcota, Proteobacteria, Crenarchaeota, Planctomycetota, and Actinobacteriota. For fungi, the dominant phyla with higher richness included Glomeromycota, Chytridiomycota, Calcarisporiellomycota, Mortierellomycota, Zoopagomycota, Ascomycota, Rozellomycota, and Basidiomycota. The composition of soil microbial communities varied significantly across the four stages. Both bacterial and fungal ASVs were most abundant in the NRD stage, and dominant species differed notably among these stages (Figure 2b,d).

**FIGURE 3:**
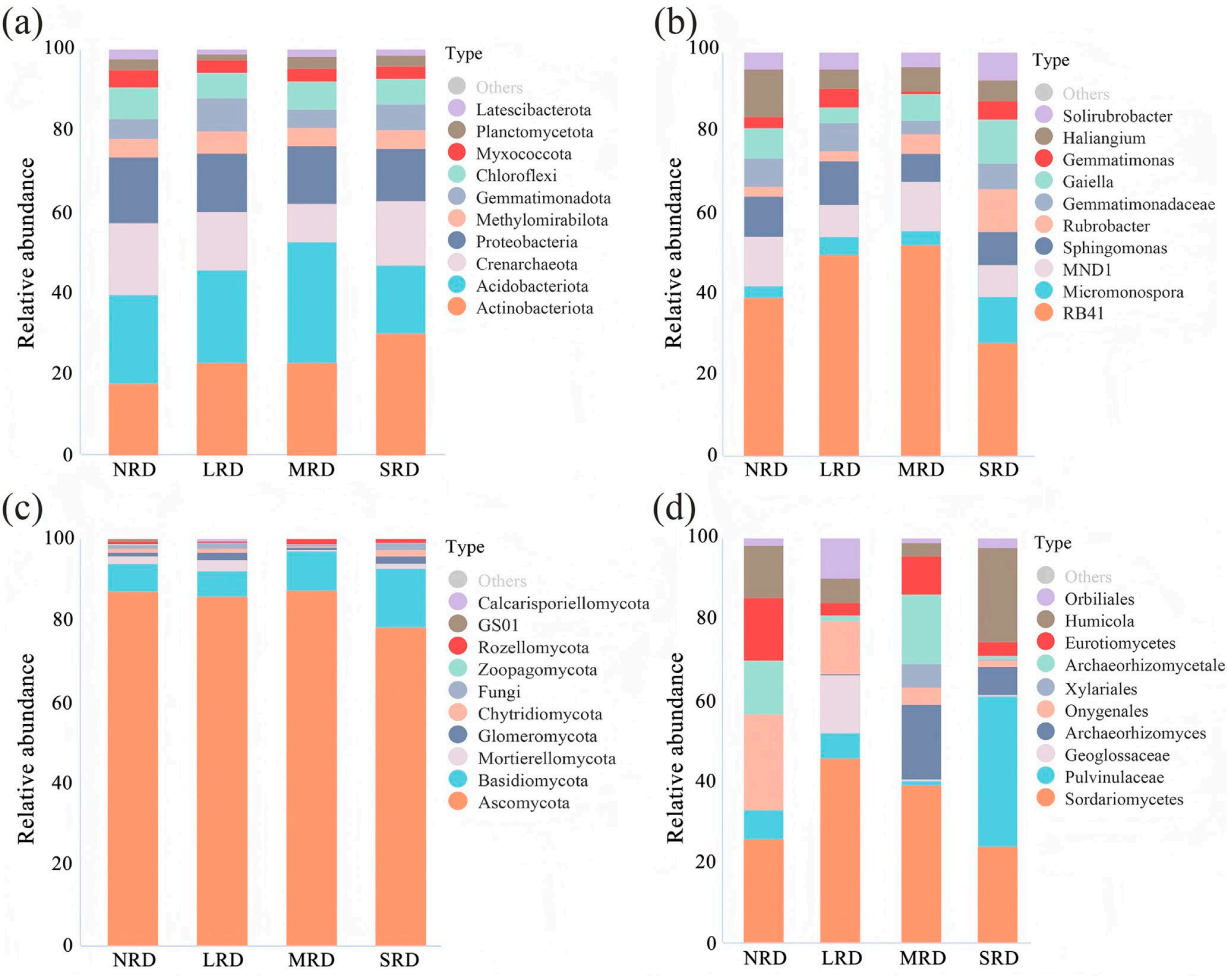
Relative abundance of soil bacteria (a, b) and fungi (c, d) at the phylum and genus level, respectively, in the different KRD stages. KRD, karst rocky desertification; LRD, light rocky desertification; MRD, moderate rocky desertification; NRD, nil rocky desertification; SRD, severe rocky desertification.

The species richness of dominant bacterial and fungal taxa exhibited distinct variations across the four stages, showing clear coupling with desertification severity. At the bacterial phylum level (Figure 3a): With intensifying desertification, the relative abundance of Actinobacteriota (16.22%–28.01%) gradually increased, the relative abundance of Acidobacteriota (20.3%–15.8%) displayed an initial increase followed by a decrease, and the relative abundance of Crenarchaeota (16.21%–14.68%) first decreased and then rebounded with increasing desertification. At the bacterial genus level (Figure 3b): Along the desertification gradient, the relative abundance of RB41 (4.9%–4.17%) showed an initial rise followed by a decline, and the relative abundance of Micromonospora (0.38%–1.7%) consistently increased along the desertification gradient. At the fungal phylum level (Figure 3c): the relative abundance of Ascomycota exceeded 50% across all four rocky desertification stages, peaking at 67.94% with a minimum of 56.83%. The relative abundance of Basidiomycota (1.36%–10.51%) first decreased then increased with increasing desertification severity. At the fungal genus level (Figure 3d), taxa showed weaker correlations with the desertification degree succession process.

### 3.2 Soil Microbial Diversity

**FIGURE 4:**
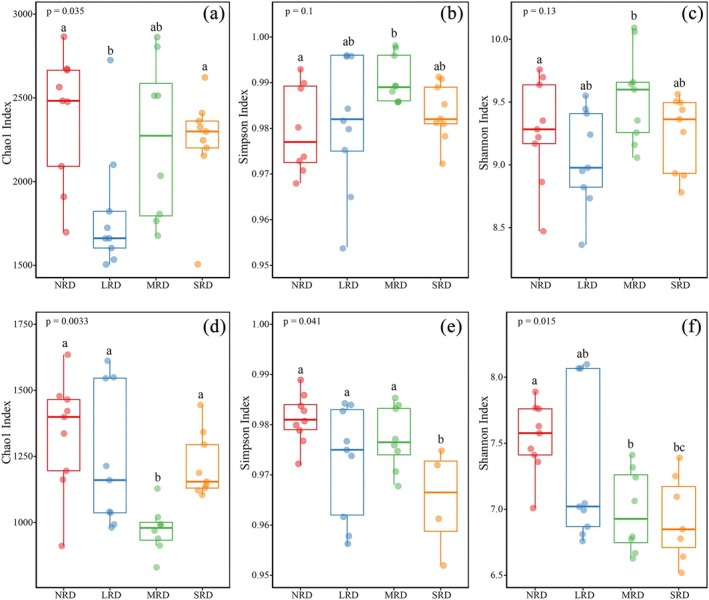
α‐Diversity indexes of soil bacteria (a, b, c) and fungi (d, e, f) in the different KRD stages. KRD, karst rocky desertification; LRD, light rocky desertification; MRD, moderate rocky desertification; NRD, nil rocky desertification; SRD, severe rocky desertification. No same letter represents the significant (*p* < 0.05) differences in the same diversity index between KRD stages.

Whether for soil bacteria or fungi, the Chao1, Simpson, and Shannon diversity indices showed significant (*p* < 0.05) differences across various rocky desertification stages. The soil bacterial Chao1 index was the lowest in LRD (Figure 4a) and exhibited a trend of initial decline followed by recovery with intensifying desertification. The soil bacterial Simpson and Shannon indices in MRD were significantly (*p* < 0.05) higher than those in NRD (Figure 4b,c), both indices first rose and then declined along the desertification gradient. The soil fungal Chao1 index followed a similar trend to that of bacteria, decreasing initially and then recovering with desertification intensified (Figure 4d), though the lowest value occurred in MRD. The fungal Simpson index decreased progressively with increasing desertification severity, reaching its lowest point in SRD (Figure 4e). Similarly, the fungal Shannon index also displayed an overall declining trend along the same gradient (Figure 4f).

**FIGURE 5:**
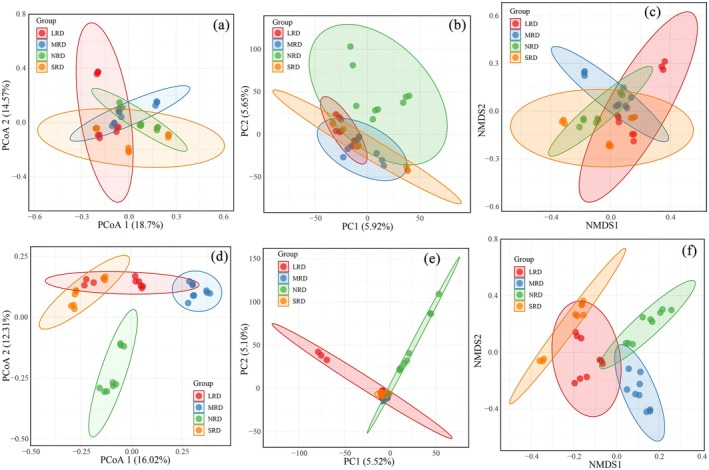
β‐Diversity indexes of soil bacteria (a, b, c) and fungi (d, e, f) in the different KRD stages. KRD, karst rocky desertification; LRD, light rocky desertification; MRD, moderate rocky desertification; NRD, nil rocky desertification; SRD, severe rocky desertification.

PCoA and NMDS analyses of soil bacterial communities showed that the point clusters representing bacterial communities were spatially separated across the four stages, but they exhibited substantial overlap (Figure 5a,c), indicating no significant (*p* < 0.05) differences in bacterial community structure among them. However, PCA analysis of bacterial communities showed that the composition of bacterial communities in the NRD stage differed significantly from the other three stages (Figure 5b). PCoA analysis of soil fungal communities (Figure 5d) demonstrated that the fungal community structure in NRD was significantly distinct from the other three rocky desertification degrees, while MRD and SRD also exhibited significant differences between them. However, the fungal community structure in LRD overlapped considerably with both that of SRD and MRD. PCA analysis of fungal communities (Figure 5e) indicated extensive overlap across the four stages, suggesting no clear structural differences among them. In contrast, the fungal NMDS analysis (Figure 5f) revealed distinct differences in community structure along the desertification gradient, with significant divergence observed between the fungal communities of NRD and MRD compared to SRD. Compared to soil bacterial communities, soil fungal communities displayed more pronounced structural differences across varying desertification degrees.

### 3.3 Plant Functional Diversity

**FIGURE 6:**
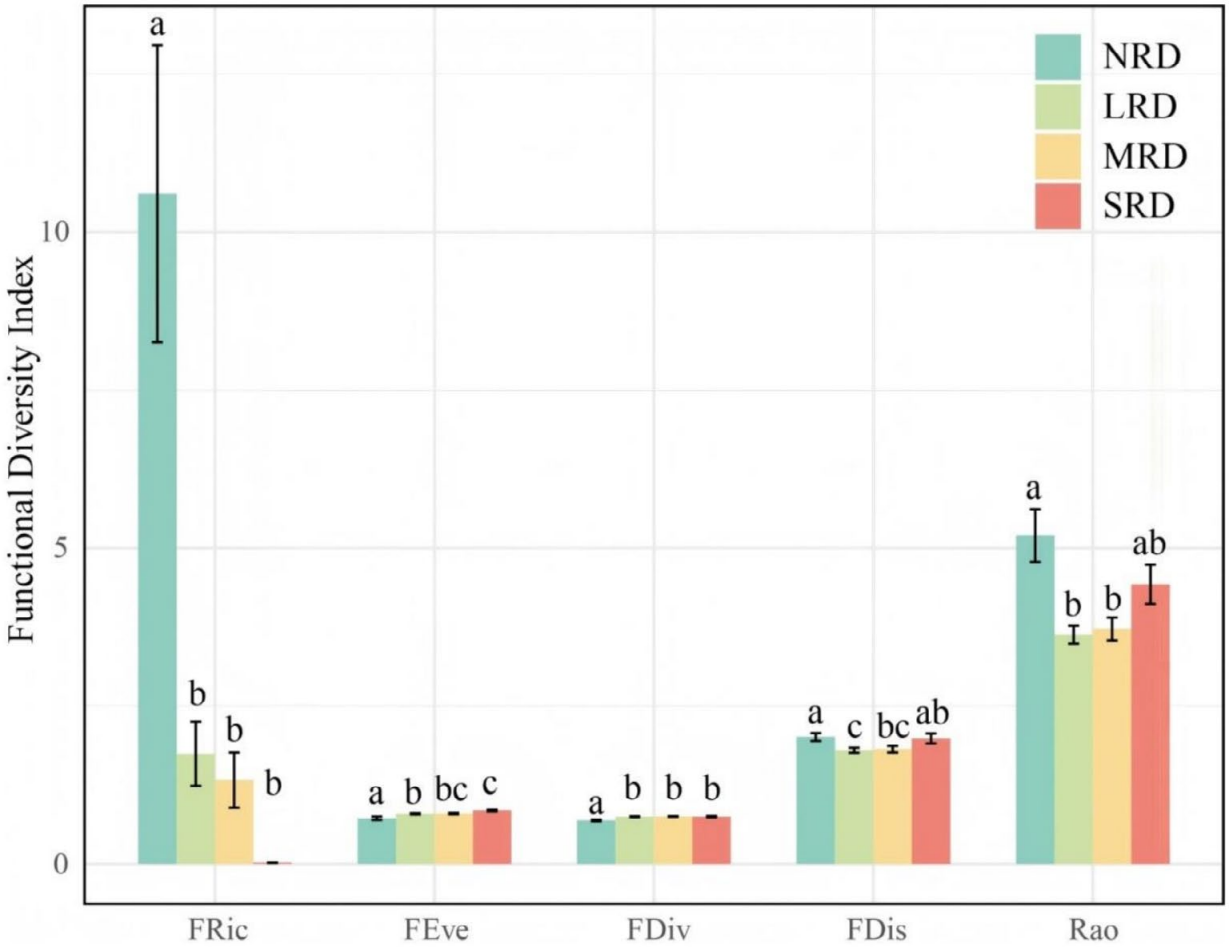
Plant functional diversity in the different KRD stages. FDis, functional dispersion index; FDiv, functional divergence index; FEve, functional uniformity index; FRic, functional richness index; KRD, karst rocky desertification; LRD, light rocky desertification; MRD, moderate rocky desertification; NRD, nil rocky desertification; Rao, Rao's quadratic entropy; SRD, severe rocky desertification. No same letter represents the significant (*p* < 0.05) differences in the same functional diversity index between KRD stages.

The five plant functional diversity indices exhibited significant (*p* < 0.05) differences across the four desertification stages (Figure 6). The functional richness index (FRic) was significantly highest in NRD, markedly exceeding that of the other three stages. With desertification intensification, the FRic index showed a gradual declining trend. In contrast, the functional evenness index (FEve) and functional divergence index (FDiv) in NRD were significantly lower than those in the other stages. Along the desertification gradient, the FEve index displayed a progressive upward trend. The functional dispersion index (FDis) and Rao's quadratic entropy index (Rao) in NRD were significantly higher than those in LRD and MRD, but showed no significant difference compared to those in SRD. Both indices of FDis and Rao exhibited a decline‐then‐rise pattern with increasing desertification severity.

### 3.4 Correlation Analysis: Relationships Between Soil Microbes and Soil Physicochemical Properties/Plant Functional Diversity

**FIGURE 7:**
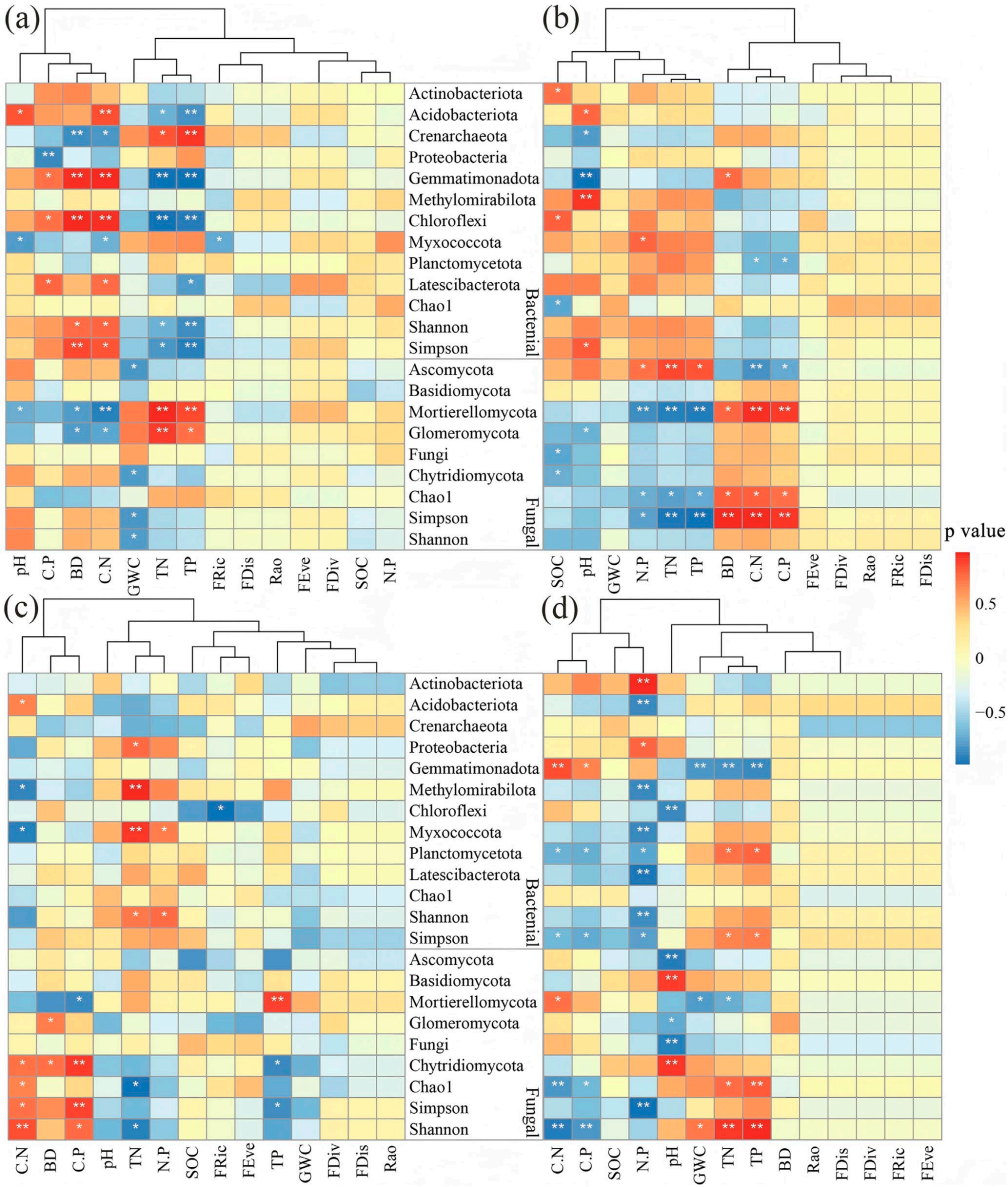
Spearman correlation of soil bacterial and fungal relative abundance and diversity in phylum level with soil physical–chemical properties and plant functional diversity in the different KRD stages of NRD (a), LRD (b), MRD (c), and SRD (d). BD, bulk density; C:N, SOC:TN; C:P, SOC:TP; FDis, functional dispersion index; FDiv, functional divergence index; FEve, functional uniformity index; FRic, functional richness index; KRD, karst rocky desertification; LRD, light rocky desertification; MRD, moderate rocky desertification; N:P, TN:TP; NRD, nil rocky desertification; Rao, Rao's quadratic entropy; SOC, soil organic carbon content; SRD, severe rocky desertification; SWC, soil water content; TN, total nitrogen content; TP, total phosphorus content. * and ** represent the significant correlation at *α* = 0.05 and 0.01, respectively.

Correlation analysis was employed to clarify the specific patterns and strengths of the associations between soil microbial communities (bacteria and fungi) and various indicators of soil physicochemical properties and plant functional diversity across different rocky desertification stages (Figure 7). The analysis revealed significant (*p* < 0.05) or highly significant (*p* < 0.01) correlations between the composition and diversity indices of microbial communities and multiple soil physicochemical factors and plant functional diversity. However, the strength and pattern of these correlations varied significantly with the degree of desertification.

In the NRD stage (Figure 7a), soil bacterial communities significantly (*p* < 0.05) or highly significantly (*p* < 0.01) correlated with soil BD, C:N, TP, TN, C:P, pH, and plant FRic index; and soil fungal communities significantly or highly significantly correlated with soil pH, BD, TN, C:N, GWC, and TP. In LRD stage (Figure 7b), soil bacterial communities showed significant or highly significant correlations with soil SOC, pH, N:P, BD, C:N, and C:P; and soil fungal communities significantly or highly significantly correlated with SOC, pH, N:P, TN, TP, BD, C:N, and C:P. In MRD stage (Figure 7c), soil bacterial communities associated significantly or highly significantly with soil C:N, TN, N:P, and plant FRic index; and soil fungal communities correlated significantly or highly significantly with soil C:N, BD, C:P, TN, and TP. In SRD stage (Figure 7d), both soil bacterial and fungal communities demonstrated significant or highly significant correlations with soil C:N, C:P, N:P, pH, GWC, TN, and TP.

Among the five plant functional diversity indices, only the FRic index showed significant (*p* < 0.05) correlations with bacterial abundance in NRD and MRD stages. Overall, soil physicochemical properties exhibited significantly stronger correlations with microbial communities than plant functional diversity did.

### 3.5 Mechanistic Insights: Plant–Soil‐Microbe Interactions During Rocky Desertification Succession

Redundancy analysis (RDA) and partial least squares structural equation modeling (PLS‐SEM) were conducted to reveal the interaction pathways and driving mechanisms among soil microbial communities, soil physicochemical properties, and plant functional diversity during rocky desertification succession.

**FIGURE 8:**
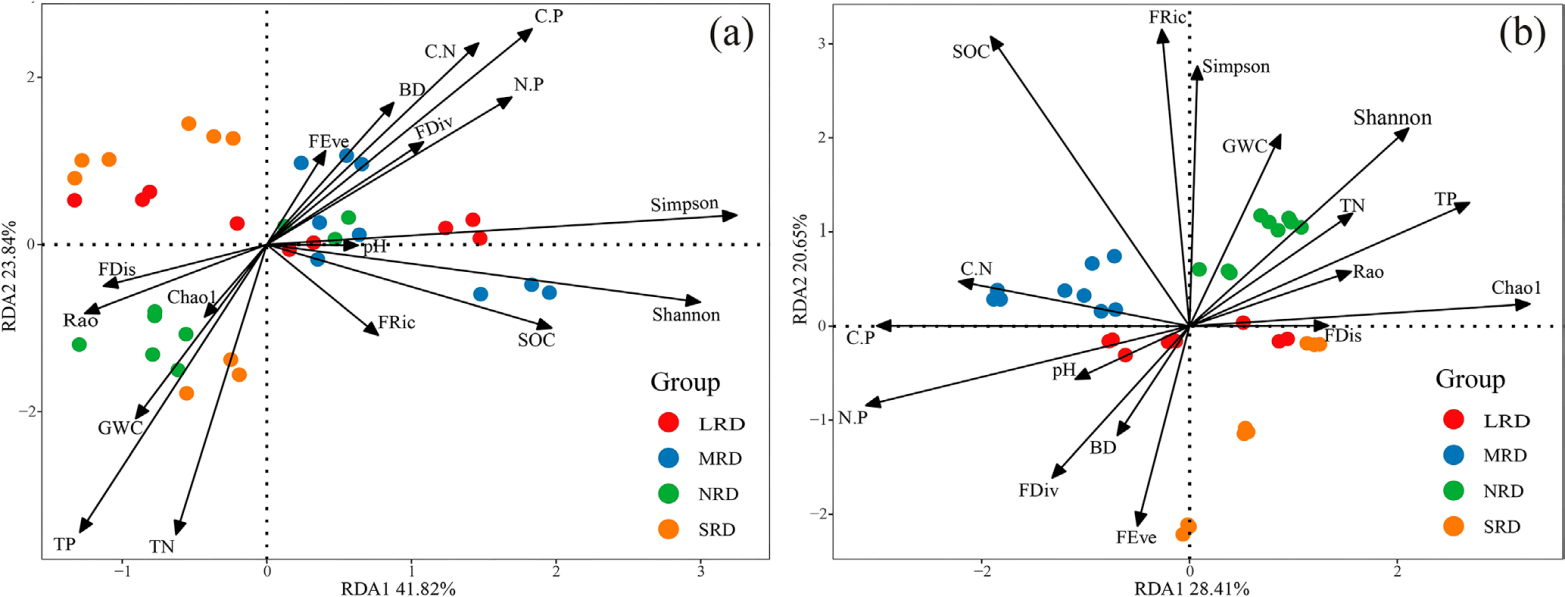
Redundancy analysis (RDA) for the relationships of soil bacteria (a) and fungi (b) community with soil physical–chemical properties and plant functional diversity in the karst rocky desertification ecosystem. BD, bulk density; C:N, SOC:TN; C:P, SOC:TP; FDis, functional dispersion index; FDiv, functional divergence index; FEve, functional uniformity index; FRic, functional richness index; LRD, light rocky desertification; MRD, moderate rocky desertification; N:P, TN:TP; NRD, nil rocky desertification; Rao, Rao's quadratic entropy; SOC, soil organic carbon content; SRD, severe rocky desertification; SWC, soil water content; TN, total nitrogen content; TP, total phosphorus content.

The RDA results (Figure 8) quantified the contributions of key driving factors and their stage‐specific variations. For bacterial communities, the first two axes (RDA1 and RDA2) together explained 67.27% of the total variation (Figure 8a); for fungal communities, they explained 55.06% (Figure 8b). In Figure 8a, arrows of soil SOC, C:P, N:P, and C:N were long and had acute angles with bacterial Simpson and Shannon indices; arrows of soil TN and TP were long and had acute angles with bacterial Chaol index, indicating these factors had strong effects on bacterial communities. In Figure 8b, arrows of soil TP were long and had acute angles with fungal Chaol and Shannon indices; arrows of SOC and plant FRic index were long and had acute angles with fungal Simpson index; arrows of soil C:P and N:P were long and had obtuse angles with fungal indices, indicating these factors had strong effects on fungal communities. In addition, the analysis identified that the key factors shaping microbial community structure shifted distinctly across succession stages: soil GWC and TP were key in the NRD stage; soil pH, FDis index, and soil N:P in the LRD stage; soil SOC and N:P in the MRD stage; and soil TN and FDis index in the SRD stage.

**FIGURE 9:**
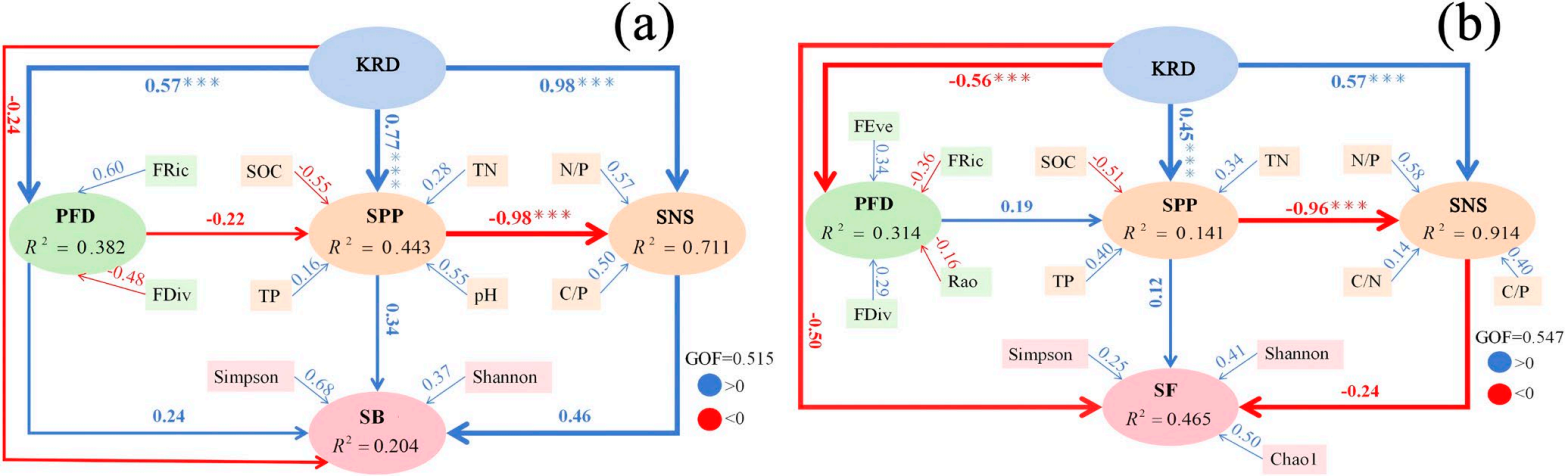
Relationships among soil physical–chemical properties (SPP), plant functional diversity (PFD), soil nutrient stoichiometry (SNS), soil bacteria (SB) (a) or fungi (SF) (b) community in karst rocky desertification evolution (KRD) by the model of PLS‐SEM. Blue and red arrows represent the positive and negative correlation, respectively. The number near the arrow is the path coefficient. *R* ^2^ represents the proportion of variance explained by each dependent variable. *** indicates the significant difference (*p* < 0.01).

The PLS‐SEM analysis (Figure 9) further elucidated the direct and indirect regulatory pathways among these components. The model indicated that soil microbial diversity decreased with increasing desertification severity, but the underlying regulatory mechanisms differed: Soil bacterial diversity was directly influenced by both plant functional diversity and soil physicochemical properties (Figure 9a). Plant FRic and FDiv, soil SOC, N:P, C:P, and pH were the main factors influencing bacterial Simpson and Shannon indices. Whereas, soil fungal diversity was primarily directly regulated by soil physicochemical properties, with plant functional diversity exerting an indirect effect mediated through its influence on soil properties (Figure 9b). Plant FRic and FEve, soil SOC, TP, N:P, and C:P were the main factors influencing fungal Chaol and Shannon indices.

## 4 Discussion

### 4.1 Changes in Soil Microbial Community Composition and Species Diversity During Karst Rocky Desertification Succession

During the karst rocky desertification succession, soil microbial community structure underwent significant changes, with bacteria exhibiting more pronounced shifts than fungi (Figures 2 and 3). At the soil bacteria phylum level, Actinobacteriota and Acidobacteriota remained dominant groups across all rocky desertification succession stages (Figure 3a). Actinobacteriota, known for their prevalence in nutrient‐poor soils and remarkable adaptability to low organic carbon conditions (Fierer et al. 2007). The present study results, consistent with this conclusion, showed relative abundance of Actinobacteriota increased along with the desertification gradient, peaking in SRD. The phylum Acidobacteriota demonstrates strong adaptability to acidic soils, with research indicating its typically abundant presence in low‐pH environments (Rousk et al. 2009). Studies have shown that fluctuations in soil pH may significantly influence its relative abundance (Rousk et al. 2009). Contrary to these results, our study revealed their persistent dominance in weakly alkaline rocky desertification environments. Soil fungal communities were dominated by Ascomycota (> 50% relative abundance across all stages) in the karst rocky desertification environments, though their abundance progressively decreased with intensifying rocky desertification, reaching minimum levels in SRD (Figure 3c). This reduction likely reflects diminished plant roots and consequent nutrient limitation in advanced degradation stages of rocky desertification (Challacombe et al. 2019; Zuo et al. 2024). The phylum Basidiomycota exhibits strong dependence on plant root exudates and soil organic matter (Meng et al. 2024). The present study results showed a U‐shaped trend in Basidiomycota relative abundance along the rocky desertification gradient, that is, initially decreasing but subsequently recovering with increasing degradation severity. This pattern suggests that soil organic matter content does not always decline but rather shows initial decrease followed by recovery during rocky desertification progression, consistent with the findings of Sheng et al. (2018).

With intensifying karst rocky desertification, both soil bacterial and fungal communities exhibited significant declines in Chao1 and Shannon indices, while the Simpson index remained relatively stable (Pei et al. 2024). This indicates that the dominant microbial populations showed no substantial changes despite increasing desertification severity. During the rocky desertification succession, soil bacterial diversity demonstrated an unobvious reduction pattern, though a clear decrease was observed in SRD compared to NRD (Figure 4a–c). Fungal diversity and species abundance showed a marked progressive decline with rocky desertification degree increasing (Figures 4 and 5), consistent with the study results by Zheng et al. (2024). Studies by He et al. (2019) similarly showed that karst rocky desertification succession significantly changed surface vegetation and obviously altered soil fungal composition and diversity, consequently resulting in substantial variations in soil fungal community structure. Rocky desertification induces a triad of soil degradation effects: (1) reduction in organic matter content, (2) elevation of pH levels, and (3) depletion of soil nutrients. These altered edaphic conditions directly compromise soil microbial habitats, ultimately driving decreased biodiversity (Zhang et al. 2023). When karst rocky desertification happens, vegetation degradation reduces soil organic matter input, thereby limiting microbial food sources and ultimately decreasing microbial diversity (Zhang et al. 2023); and the reduction in both litter variety and the quantity/diversity of plant root exudates further contributes to diminished soil microbial diversity (Bardgett and van der Putten 2014).

In summary, karst rocky desertification leads to obvious vegetation degradation, severe water and soil loss, and serious land productivity loss, consequently altering soil microbial community structure and diversity. During karst rocky desertification succession, both bacterial and fungal communities demonstrated significant response patterns in terms of structure composition and diversity indices, thereby validating the first scientific hypothesis of the present study.

### 4.2 Driving Factors of Soil Microbial Community Changes During Karst Rocky Desertification Succession

Changes in soil organic matter, nitrogen, phosphorus, and other nutrient contents directly affect microbial growth and metabolism (Fierer and Jackson 2006; Liu et al. 2018), inevitably leading to significant alterations in soil microbial diversity and species composition (Zhou, Jia, et al. 2020; Zhou, Wang, and Luo 2020; Guo et al. 2021). Consistent with this conclusion, the present study demonstrates that soil C:N ratio, N:P ratio, TP, and SOC serve as key factors influencing soil bacterial diversity and community structure (Figures 7, 8, 9). Soil pH emerges as a primary driver of microbial diversity and compositional shifts (Liu et al. 2018; Zhou, Jia, et al. 2020; Zhou, Wang, and Luo 2020). Bacterial communities in acidic soils (pH 5.2) differ significantly from those in neutral to weakly alkaline soils (pH 7.7) (Cho et al. 2016), with pH exerting stronger effects on soil bacterial communities than spatial or climatic factors. Most bacterial taxa exhibit alkaline tolerance (Lu et al. 2023; Zhou et al. 2024). Consistent with these conclusions, the present study showed that pH significantly shapes soil bacterial community composition and diversity. Furthermore, fungal community composition and diversity showed significant positive correlations with soil C:N, C:P, TP, and TN (Figure 7), confirming that soil physicochemical properties also strongly affected fungal communities (Jiao et al. 2021). In summary, soil physicochemical properties act as direct drivers of bacterial and fungal community responses during karst rocky desertification succession. As the karst rocky desertification progresses, vegetation reductions induce marked shifts in soil pH, organic carbon, total nitrogen, and moisture content, which in turn directly mediate microbial community restructuring.

Aboveground vegetation characteristics, including plant species composition, genetic diversity, root system, and growth status, exert profound influences on soil microbial communities (Lei et al. 2016; Zhou, Jia, et al. 2020; Zhou, Wang, and Luo 2020). Pan et al. (2021) demonstrated that plant functional traits and their diversity may indirectly regulate soil microbial community functionality and diversity. Consistent with this deduction, the present study revealed that the aboveground plant functional diversity has significant effects on soil microbial diversity and community structure (Figure 9). The FRic index of aboveground plants showed a significant positive correlation with soil bacterial diversity (Figure 9a), while the FEve and FDiv indices exhibited significant positive correlations with soil fungal diversity (Figure 9b). So, the second hypothesis proposed in the present study, that is, during the rocky desertification succession process, changes in soil microorganisms exhibit a significant correlation with the functional diversity of aboveground plants, is confirmed. Bardgett and van der Putten (2014) also highlighted that plant functional traits exhibit significant correlations with the community composition and functional traits of soil fungi, particularly in the rhizosphere microhabitat. Changes in plant functional diversity directly influence soil physicochemical properties, thereby directly or indirectly affecting the community composition and diversity of soil microorganisms (Figure 9). Plant communities with richer functional traits significantly increase fungal species richness by providing diverse carbon sources (Boeddinghaus et al. 2019). Plant functional traits may have an ameliorative effect on the soil microenvironment, and plant communities with higher FEve usually exhibit greater litter input. In summary, with the occurrence and progressive intensification of karst rocky desertification, the aboveground plant communities undergo significant degradation, leading to a marked decline in plant functional diversity. These changes indirectly drive alterations in soil microbial diversity and community structure by modifying soil physicochemical properties. During this process, the FRic, FEve, and FDiv indices of aboveground plants play crucial roles. During the rocky desertification succession process, there are very complex interactions present among plants, soils, and microorganisms. Elucidating these relationships is of great significance for vegetation restoration and rocky desertification control in karst regions, which warrants further in‐depth studies in the future.

## 5 Conclusions

In the karst rocky desertification ecosystem, main taxa of soil bacteria were Gemmatimonadota, Methylomirabilota, Acidobacterota, Latescibacterota, Chloroflexi, Myxococcota, Proteobacteria, Crenarchaeota, Planctomycetota, and Actinobacteriota; and main taxa of soil fungi were Glomeromycota, Chytridiomycota, Calcarisporiellomycota, Mortierellomycota, Zoopagomycota, Ascomycota, Rozellomycota, and Basidiomycota. The composition of soil microbial communities and diversity varied significantly (*p* < 0.05) across different rocky desertification degrees. Compared to soil bacterial communities, soil fungal communities displayed more pronounced structural differences across varying rocky desertification degrees. Functional diversity of aboveground plants also exhibited significant (*p* < 0.05) differences across varying rocky desertification degrees. Both taxonomic composition and diversity indices of soil microorganisms (bacteria and fungi) showed significant (*p* < 0.05) or highly significant (*p* < 0.01) correlations with soil physicochemical properties and plant functional diversity across the four rocky desertification degrees. The two scientific hypotheses proposed in the present study were confirmed. During karst rocky desertification succession, both bacterial and fungal communities demonstrated significant response patterns in terms of structural composition and diversity indices; and changes in soil microorganisms exhibit a significant correlation with the functional diversity of aboveground plants. During the karst rocky desertification succession, soil bacterial diversity was directly influenced by plant functional diversity and soil physicochemical properties. While soil fungal diversity exhibited direct regulation by soil physicochemical properties and indirect effects from plant functional diversity mediated through soil properties. Constrained by the conditions of field experiments and the time‐sensitive nature of samples, this study was unable to directly obtain root exudate data, affecting the establishment of the relationship between plant and microbe. In subsequent studies, directly determining plant root exudates to further verify and refine the relationship between plants and microorganisms is important and necessary. During the rocky desertification succession process, there are very complex interactions present among plants, soils, and microorganisms. Elucidating these relationships is of great significance for vegetation restoration and rocky desertification control in karst regions, which warrants further in‐depth studies in the future.

## Author Contributions

**Dandan Zhu:** data curation (lead), formal analysis (lead), investigation (lead), software (lead), visualization (lead), writing – original draft (equal). **Maoyin Sheng:** conceptualization (equal), methodology (lead), project administration (lead), resources (equal), supervision (lead), validation (equal), writing – original draft (supporting), writing – review and editing (lead). **Yaoyao Liu:** data curation (supporting), formal analysis (supporting), investigation (supporting), software (supporting), validation (supporting), visualization (supporting), writing – original draft (supporting). **Linjiao Wang:** conceptualization (equal), funding acquisition (equal), methodology (equal), project administration (supporting), resources (equal), supervision (supporting), writing – review and editing (supporting).

## Funding

This work was supported by the Talent Scientific Research Foundation of Hefei Institute of Technology (No. 2025KY55); Natural Science Foundation of Guizhou Province (Qiankehe Zhongyindi [2023]028); and Science and Technology Program of Guizhou Province (Qainkehe Zhicheng [2024]yiban120).

## Conflicts of Interest

The authors declare no conflicts of interest.

## Supporting information


**Table S1:** Basic information of sample plots in the study.
**Table S2:** Soil physical and chemical properties of the sample plots.
**Table S3:** Relative abundance of the top 10 bacterial taxa (phylum) of the sample plots.
**Table S4:** Relative abundance of the top 10 fungal taxa (phylum) of the sample plots.
**Table S5:** Soil microbial α diversity indexes of the sample plots.
**Table S6:** Coordinate data for PCoA, PCA, and NMDS (bacterial).
**Table S7:** Coordinate data for PCoA/PCA/NMDS (fungal).
**Table S8:** Plant functional traits and functional diversity indices of the sample plots.

## Data Availability

The data that supports the findings of this study are available in Supporting Information Table S1–S8 of this article.
