# Supplementary material for: Responses of Soil Microbial Community to Rocky Desertification Succession and Their Relationships With Plant Functional Diversity in Southwest China Karst Ecosystem
Source: Ecol Evol. 2026 Jan 30;16(2):e73013. doi: 10.1002/ece3.73013 (PMC12859167; doi:10.1002/ece3.73013)
Supplement: Supplementary file 1 — Table S1: Basic information of sample plots in the study. Table S2: Soil physical and chemical properties of the sample plots. Table S3: Relative abundance of the top 10 bacterial taxa (phylum) of the sample plots. Table S4: Relative abundance of the top 10 fungal taxa (phylum) of the sample plots. Table S5: Soil microbial α diversity indexes of the sample plots. Table S6: Coordinate data for PCoA, PCA, and NMDS (bacterial). Table S7: Coordinate data for PCoA/PCA/NMDS (fungal). Table S8: Plant functional traits and functional diversity indices of the sample plots. [file ECE3-16-e73013-s001.docx]

**Supplementary Data**

**Contents:**

Table S1: Basic information of sample plots in the study.

Table S2: Soil physical and chemical properties of the sample plots.

Table S3: Relative abundance of the top 10 bacterial taxa (Phylum) of the sample plots.

Table S4: Relative abundance of the top 10 fungal taxa (Phylum) of the sample plots.

Table S5: Soil microbial α diversity indexes of the sample plots.

Table S6: Coordinate data for PCoA, PCA, and NMDS (Bacterial).

Table S7: Coordinate data for PCoA/PCA/NMDS (Fungal).

Table S8: Plant functional traits and functional diversity indices of the sample plots.

**Table S1**

Basic information of sample plots in the study.

| No. of sample plot | Karst rocky desertification degree | Altitude  (m) | Aspect of slope | Slope  position | Slope  (°) | Soil type | Percentage of  exposed rock  (%) | Percentage of  vegetation cover  (%) |
| --- | --- | --- | --- | --- | --- | --- | --- | --- |
| Nrd-1 | Nil | 839 | Northwest | Middle | 9 | Yellow soil | 20 | 90 |
| Nrd-2 | Nil | 828 | Southwest | Middle | 7 | Yellow soil | 30 | 85 |
| Nrd-3 | Nil | 834 | North | Middle | 8 | Yellow soil | 28 | 87 |
| Lrd-1 | Light | 708 | Northeast | Middle | 9 | Yellow soil | 40 | 71 |
| Lrd-2 | Light | 712 | East | Middle | 10 | Yellow soil | 38 | 70 |
| Lrd-3 | Light | 710 | East | Middle | 10 | Yellow soil | 32 | 72 |
| Mrd-1 | Moderate | 835 | Southwest | Middle | 12 | Yellow soil | 75 | 25 |
| Mrd-2 | Moderate | 827 | Southwest | Middle | 10 | Yellow soil | 70 | 30 |
| Mrd-3 | Moderate | 865 | Northwest | Middle | 14 | Yellow soil | 72 | 27 |
| Srd-1 | Severe | 761 | Northwest | Middle | 15 | Yellow soil | 90 | 12 |
| Srd-2 | Severe | 767 | Northwest | Middle | 11 | Yellow soil | 89 | 13 |
| Srd-3 | Severe | 775 | Northeast | Middle | 13 | Yellow soil | 85 | 15 |

**Table S2**

Soil physical and chemical properties of the sample plots.

| Sample plot | SOC  (g·kg^-1^) | TN  (g·kg^-1^) | TP  (g·kg^-1^) | pH | BD  (%) | GWC  (%) | C/N | C/P | N/P |
| --- | --- | --- | --- | --- | --- | --- | --- | --- | --- |
| Nrd-1 | 36.36 | 1.64 | 0.97 | 7.35 | 1.04 | 33.17 | 22.17 | 37.62 | 1.70 |
| Nrd-1 | 35.52 | 1.55 | 1.04 | 7.44 | 1.01 | 36.59 | 22.91 | 34.16 | 1.49 |
| Nrd-1 | 35.09 | 1.62 | 1.06 | 7.38 | 1.17 | 24.54 | 21.66 | 33.05 | 1.53 |
| Nrd-2 | 35.52 | 2.37 | 1.28 | 7.41 | 0.90 | 35.27 | 14.99 | 27.67 | 1.85 |
| Nrd-2 | 30.02 | 2.42 | 1.79 | 7.42 | 0.87 | 22.62 | 12.41 | 16.82 | 1.36 |
| Nrd-2 | 38.48 | 2.27 | 1.34 | 7.42 | 0.85 | 29.39 | 16.95 | 28.82 | 1.70 |
| Nrd-3 | 30.44 | 1.30 | 0.93 | 7.43 | 1.29 | 10.36 | 23.42 | 32.91 | 1.41 |
| Nrd-3 | 38.05 | 1.41 | 0.88 | 7.45 | 1.14 | 11.12 | 26.99 | 43.19 | 1.60 |
| Nrd-3 | 32.56 | 1.34 | 0.88 | 7.46 | 1.24 | 13.00 | 24.30 | 36.95 | 1.52 |
| Lrd-1 | 31.29 | 1.40 | 0.76 | 7.33 | 1.28 | 17.11 | 22.35 | 41.36 | 1.85 |
| Lrd-1 | 32.56 | 1.40 | 0.76 | 7.46 | 1.05 | 13.75 | 23.26 | 42.62 | 1.83 |
| Lrd-1 | 32.98 | 1.37 | 0.75 | 7.35 | 1.20 | 14.05 | 24.07 | 44.02 | 1.83 |
| Lrd-2 | 31.29 | 1.60 | 0.83 | 7.29 | 1.15 | 22.39 | 19.56 | 37.71 | 1.93 |
| Lrd-2 | 30.87 | 1.62 | 0.88 | 7.40 | 0.97 | 20.96 | 19.05 | 35.03 | 1.84 |
| Lrd-2 | 32.98 | 1.58 | 0.84 | 7.38 | 1.14 | 10.06 | 20.87 | 39.40 | 1.89 |
| Lrd-3 | 29.60 | 1.09 | 0.63 | 7.30 | 1.32 | 18.22 | 27.15 | 46.65 | 1.72 |
| Lrd-3 | 30.02 | 1.12 | 0.69 | 7.28 | 1.40 | 11.67 | 26.80 | 43.47 | 1.62 |
| Lrd-3 | 31.29 | 1.08 | 0.71 | 7.25 | 1.24 | 9.88 | 28.97 | 44.21 | 1.53 |
| Mrd-1 | 34.67 | 1.61 | 0.68 | 7.59 | 0.92 | 33.80 | 21.53 | 50.74 | 2.36 |
| Mrd-1 | 38.05 | 1.55 | 0.71 | 7.56 | 0.88 | 36.35 | 24.55 | 53.59 | 2.18 |
| Mrd-1 | 35.94 | 1.51 | 0.69 | 7.56 | 0.88 | 42.25 | 23.80 | 52.23 | 2.19 |
| Mrd-2 | 36.36 | 1.13 | 0.55 | 7.54 | 1.26 | 14.94 | 32.18 | 66.52 | 2.07 |
| Mrd-2 | 38.90 | 1.23 | 0.52 | 7.58 | 1.22 | 12.97 | 31.63 | 74.61 | 2.36 |
| Mrd-2 | 32.13 | 1.23 | 0.56 | 7.56 | 1.24 | 14.20 | 26.13 | 57.01 | 2.18 |
| Mrd-3 | 37.63 | 1.62 | 0.69 | 7.56 | 1.23 | 15.07 | 23.23 | 54.29 | 2.34 |
| Mrd-3 | 36.36 | 1.62 | 0.68 | 7.58 | 1.12 | 13.74 | 22.45 | 53.79 | 2.40 |
| Mrd-3 | 40.59 | 1.66 | 0.68 | 7.58 | 1.25 | 10.63 | 24.45 | 59.62 | 2.44 |
| Srd-1 | 24.95 | 2.84 | 2.17 | 7.55 | 1.02 | 33.32 | 8.78 | 11.51 | 1.31 |
| Srd-1 | 25.79 | 2.84 | 1.88 | 7.62 | 1.05 | 23.24 | 9.08 | 13.76 | 1.51 |
| Srd-1 | 25.79 | 2.88 | 1.91 | 7.58 | 1.20 | 15.63 | 8.96 | 13.53 | 1.51 |
| Srd-2 | 27.06 | 1.55 | 0.66 | 7.67 | 1.07 | 24.44 | 17.46 | 40.78 | 2.34 |
| Srd-2 | 25.37 | 1.62 | 0.74 | 7.64 | 1.11 | 19.72 | 15.66 | 34.08 | 2.18 |
| Srd-2 | 27.06 | 1.60 | 0.73 | 7.62 | 1.01 | 14.88 | 16.91 | 36.84 | 2.18 |
| Srd-3 | 26.21 | 1.15 | 0.67 | 7.48 | 1.35 | 8.73 | 22.80 | 38.92 | 1.71 |
| Srd-3 | 21.56 | 1.19 | 0.63 | 7.40 | 1.18 | 6.43 | 18.12 | 34.39 | 1.90 |
| Srd-3 | 26.21 | 1.26 | 0.63 | 7.46 | 1.01 | 15.26 | 20.81 | 41.64 | 2.00 |

**Table S3**

Relative abundance of the top 10 bacterial taxa (Phylum) of the sample plots.

| Sample plot | Actinobacteriota | Acidobacteriota | Crenarchaeota | Proteobacteria | Gemmatimonadota | Methylomirabilota | Chloroflexi | Myxococcota | Planctomycetota | Latescibacterota |
| --- | --- | --- | --- | --- | --- | --- | --- | --- | --- | --- |
| Nrd-1 | 0.203 | 0.186 | 0.157 | 0.125 | 0.048 | 0.034 | 0.073 | 0.043 | 0.023 | 0.027 |
| Nrd-1 | 0.196 | 0.196 | 0.188 | 0.127 | 0.040 | 0.034 | 0.070 | 0.036 | 0.017 | 0.019 |
| Nrd-1 | 0.206 | 0.167 | 0.181 | 0.139 | 0.039 | 0.040 | 0.081 | 0.040 | 0.016 | 0.015 |
| Nrd-2 | 0.134 | 0.188 | 0.170 | 0.191 | 0.033 | 0.045 | 0.052 | 0.048 | 0.026 | 0.018 |
| Nrd-2 | 0.106 | 0.186 | 0.275 | 0.156 | 0.026 | 0.034 | 0.049 | 0.037 | 0.026 | 0.016 |
| Nrd-2 | 0.133 | 0.178 | 0.189 | 0.185 | 0.031 | 0.050 | 0.056 | 0.044 | 0.026 | 0.018 |
| Nrd-3 | 0.186 | 0.199 | 0.082 | 0.158 | 0.076 | 0.048 | 0.092 | 0.040 | 0.020 | 0.021 |
| Nrd-3 | 0.136 | 0.301 | 0.102 | 0.120 | 0.057 | 0.038 | 0.084 | 0.028 | 0.039 | 0.024 |
| Nrd-3 | 0.158 | 0.225 | 0.110 | 0.142 | 0.069 | 0.049 | 0.098 | 0.032 | 0.025 | 0.020 |
| Lrd-1 | 0.215 | 0.268 | 0.033 | 0.124 | 0.068 | 0.047 | 0.097 | 0.030 | 0.018 | 0.017 |
| Lrd-1 | 0.282 | 0.247 | 0.031 | 0.099 | 0.049 | 0.103 | 0.058 | 0.030 | 0.012 | 0.013 |
| Lrd-1 | 0.233 | 0.269 | 0.031 | 0.119 | 0.065 | 0.080 | 0.072 | 0.029 | 0.013 | 0.014 |
| Lrd-2 | 0.248 | 0.131 | 0.143 | 0.172 | 0.085 | 0.040 | 0.065 | 0.031 | 0.016 | 0.004 |
| Lrd-2 | 0.177 | 0.275 | 0.128 | 0.137 | 0.060 | 0.054 | 0.049 | 0.027 | 0.028 | 0.004 |
| Lrd-2 | 0.207 | 0.212 | 0.137 | 0.158 | 0.073 | 0.046 | 0.061 | 0.026 | 0.020 | 0.006 |
| Lrd-3 | 0.168 | 0.217 | 0.225 | 0.137 | 0.082 | 0.034 | 0.044 | 0.021 | 0.015 | 0.003 |
| Lrd-3 | 0.196 | 0.195 | 0.190 | 0.144 | 0.098 | 0.036 | 0.046 | 0.022 | 0.019 | 0.003 |
| Lrd-3 | 0.194 | 0.133 | 0.260 | 0.142 | 0.090 | 0.033 | 0.057 | 0.026 | 0.007 | 0.001 |
| Mrd-1 | 0.252 | 0.207 | 0.111 | 0.139 | 0.045 | 0.042 | 0.072 | 0.032 | 0.016 | 0.010 |
| Mrd-1 | 0.201 | 0.304 | 0.115 | 0.107 | 0.043 | 0.051 | 0.057 | 0.024 | 0.021 | 0.013 |
| Mrd-1 | 0.192 | 0.328 | 0.122 | 0.085 | 0.032 | 0.040 | 0.060 | 0.022 | 0.037 | 0.015 |
| Mrd-2 | 0.201 | 0.354 | 0.102 | 0.100 | 0.036 | 0.028 | 0.066 | 0.019 | 0.027 | 0.016 |
| Mrd-2 | 0.228 | 0.307 | 0.103 | 0.122 | 0.039 | 0.030 | 0.059 | 0.019 | 0.027 | 0.013 |
| Mrd-2 | 0.225 | 0.248 | 0.118 | 0.126 | 0.047 | 0.033 | 0.083 | 0.026 | 0.020 | 0.010 |
| Mrd-3 | 0.215 | 0.230 | 0.033 | 0.176 | 0.042 | 0.055 | 0.066 | 0.032 | 0.038 | 0.017 |
| Mrd-3 | 0.203 | 0.250 | 0.029 | 0.172 | 0.041 | 0.059 | 0.060 | 0.036 | 0.036 | 0.017 |
| Mrd-3 | 0.187 | 0.260 | 0.055 | 0.155 | 0.039 | 0.057 | 0.062 | 0.032 | 0.034 | 0.020 |
| Srd-1 | 0.208 | 0.220 | 0.144 | 0.094 | 0.036 | 0.066 | 0.051 | 0.037 | 0.040 | 0.024 |
| Srd-1 | 0.202 | 0.203 | 0.139 | 0.109 | 0.039 | 0.070 | 0.047 | 0.042 | 0.041 | 0.020 |
| Srd-1 | 0.217 | 0.162 | 0.155 | 0.125 | 0.041 | 0.066 | 0.051 | 0.046 | 0.032 | 0.015 |
| Srd-2 | 0.380 | 0.127 | 0.148 | 0.128 | 0.055 | 0.021 | 0.043 | 0.021 | 0.016 | 0.004 |
| Srd-2 | 0.377 | 0.122 | 0.125 | 0.141 | 0.066 | 0.017 | 0.042 | 0.022 | 0.027 | 0.005 |
| Srd-2 | 0.357 | 0.116 | 0.175 | 0.133 | 0.061 | 0.022 | 0.040 | 0.019 | 0.020 | 0.003 |
| Srd-3 | 0.253 | 0.170 | 0.146 | 0.121 | 0.074 | 0.034 | 0.084 | 0.025 | 0.021 | 0.009 |
| Srd-3 | 0.260 | 0.165 | 0.147 | 0.121 | 0.080 | 0.034 | 0.084 | 0.023 | 0.017 | 0.008 |
| Srd-3 | 0.266 | 0.140 | 0.142 | 0.120 | 0.077 | 0.040 | 0.092 | 0.025 | 0.026 | 0.008 |

**Table S4**

Relative abundance of the top 10 fungal taxa (Phylum) of the sample plots.

| Sample plot | Ascomycota | Basidiomycota | Mortierellomycota | Glomeromycota | Fungi | Chytridiomycota | Zoopagomycota | Rozellomycota | GS01 | Calcarisporiellomycota |
| --- | --- | --- | --- | --- | --- | --- | --- | --- | --- | --- |
| Nrd-1 | 0.606 | 0.052 | 0.016 | 0.012 | 0.006 | 0.006 | 0.000 | 0.003 | 0.000 | 0.000 |
| Nrd-1 | 0.644 | 0.049 | 0.012 | 0.009 | 0.006 | 0.004 | 0.000 | 0.005 | 0.000 | 0.000 |
| Nrd-1 | 0.649 | 0.041 | 0.010 | 0.008 | 0.005 | 0.005 | 0.000 | 0.003 | 0.000 | 0.000 |
| Nrd-2 | 0.702 | 0.056 | 0.026 | 0.010 | 0.007 | 0.007 | 0.012 | 0.003 | 0.004 | 0.000 |
| Nrd-2 | 0.680 | 0.064 | 0.022 | 0.011 | 0.005 | 0.008 | 0.010 | 0.003 | 0.004 | 0.000 |
| Nrd-2 | 0.651 | 0.055 | 0.014 | 0.010 | 0.006 | 0.006 | 0.017 | 0.004 | 0.002 | 0.000 |
| Nrd-3 | 0.746 | 0.059 | 0.007 | 0.005 | 0.005 | 0.009 | 0.000 | 0.003 | 0.006 | 0.000 |
| Nrd-3 | 0.708 | 0.056 | 0.007 | 0.005 | 0.005 | 0.010 | 0.000 | 0.003 | 0.003 | 0.000 |
| Nrd-3 | 0.705 | 0.060 | 0.006 | 0.006 | 0.008 | 0.011 | 0.000 | 0.003 | 0.002 | 0.000 |
| Lrd-1 | 0.693 | 0.106 | 0.005 | 0.002 | 0.005 | 0.001 | 0.001 | 0.000 | 0.000 | 0.001 |
| Lrd-1 | 0.684 | 0.098 | 0.008 | 0.003 | 0.005 | 0.001 | 0.000 | 0.001 | 0.000 | 0.000 |
| Lrd-1 | 0.748 | 0.076 | 0.005 | 0.003 | 0.005 | 0.001 | 0.000 | 0.001 | 0.000 | 0.001 |
| Lrd-2 | 0.736 | 0.027 | 0.001 | 0.010 | 0.011 | 0.004 | 0.003 | 0.000 | 0.000 | 0.000 |
| Lrd-2 | 0.767 | 0.028 | 0.001 | 0.009 | 0.011 | 0.007 | 0.002 | 0.001 | 0.000 | 0.000 |
| Lrd-2 | 0.746 | 0.023 | 0.001 | 0.007 | 0.009 | 0.006 | 0.001 | 0.001 | 0.000 | 0.000 |
| Lrd-3 | 0.542 | 0.030 | 0.090 | 0.027 | 0.026 | 0.013 | 0.000 | 0.005 | 0.000 | 0.004 |
| Lrd-3 | 0.582 | 0.033 | 0.049 | 0.032 | 0.017 | 0.015 | 0.000 | 0.007 | 0.000 | 0.004 |
| Lrd-3 | 0.542 | 0.040 | 0.035 | 0.030 | 0.016 | 0.012 | 0.000 | 0.007 | 0.000 | 0.005 |
| Mrd-1 | 0.758 | 0.034 | 0.003 | 0.003 | 0.004 | 0.000 | 0.000 | 0.010 | 0.000 | 0.000 |
| Mrd-1 | 0.574 | 0.051 | 0.004 | 0.002 | 0.009 | 0.001 | 0.001 | 0.008 | 0.001 | 0.000 |
| Mrd-1 | 0.665 | 0.057 | 0.004 | 0.005 | 0.004 | 0.001 | 0.001 | 0.015 | 0.000 | 0.000 |
| Mrd-2 | 0.691 | 0.056 | 0.001 | 0.005 | 0.004 | 0.004 | 0.000 | 0.001 | 0.000 | 0.000 |
| Mrd-2 | 0.666 | 0.049 | 0.001 | 0.004 | 0.007 | 0.004 | 0.000 | 0.001 | 0.000 | 0.000 |
| Mrd-2 | 0.671 | 0.053 | 0.001 | 0.005 | 0.006 | 0.002 | 0.000 | 0.001 | 0.000 | 0.000 |
| Mrd-3 | 0.591 | 0.126 | 0.003 | 0.006 | 0.007 | 0.002 | 0.000 | 0.007 | 0.000 | 0.000 |
| Mrd-3 | 0.667 | 0.104 | 0.002 | 0.004 | 0.005 | 0.002 | 0.000 | 0.006 | 0.000 | 0.000 |
| Mrd-3 | 0.596 | 0.125 | 0.004 | 0.005 | 0.005 | 0.002 | 0.000 | 0.005 | 0.000 | 0.000 |
| Srd-1 | 0.583 | 0.033 | 0.005 | 0.012 | 0.014 | 0.005 | 0.001 | 0.012 | 0.000 | 0.000 |
| Srd-1 | 0.524 | 0.031 | 0.003 | 0.011 | 0.012 | 0.004 | 0.001 | 0.006 | 0.000 | 0.000 |
| Srd-1 | 0.616 | 0.045 | 0.005 | 0.016 | 0.017 | 0.006 | 0.001 | 0.007 | 0.000 | 0.000 |
| Srd-2 | 0.513 | 0.260 | 0.005 | 0.009 | 0.007 | 0.026 | 0.000 | 0.002 | 0.000 | 0.000 |
| Srd-2 | 0.496 | 0.255 | 0.006 | 0.009 | 0.006 | 0.025 | 0.000 | 0.002 | 0.000 | 0.000 |
| Srd-2 | 0.523 | 0.242 | 0.006 | 0.007 | 0.008 | 0.024 | 0.000 | 0.001 | 0.001 | 0.000 |
| Srd-3 | 0.607 | 0.029 | 0.020 | 0.017 | 0.019 | 0.002 | 0.000 | 0.001 | 0.000 | 0.000 |
| Srd-3 | 0.633 | 0.025 | 0.014 | 0.014 | 0.018 | 0.002 | 0.000 | 0.001 | 0.000 | 0.000 |
| Srd-3 | 0.618 | 0.025 | 0.017 | 0.016 | 0.019 | 0.002 | 0.000 | 0.003 | 0.000 | 0.001 |

**Table S5**

Soil microbial α diversity indexes of the sample plots.

| Sample plot | Bacterial | | | Fungal | | |
| --- | --- | --- | --- | --- | --- | --- |
|  | Chao1 | Shannon | Simpson | Chao1 | Simpson | Shannon |
| Nrd-1 | 2091.252 | 9.283 | 0.98 | 910.872 | 0.972 | 7.008 |
| Nrd-1 | 1697.374 | 8.864 | 0.971 | 1195.802 | 0.977 | 7.359 |
| Nrd-1 | 2482.414 | 9.22 | 0.973 | 1162.505 | 0.979 | 7.411 |
| Nrd-2 | 2674.136 | 9.352 | 0.974 | 1465.603 | 0.983 | 7.629 |
| Nrd-2 | 2477.113 | 8.472 | 0.935 | 1635.5 | 0.981 | 7.576 |
| Nrd-2 | 2564.249 | 9.169 | 0.968 | 1478.39 | 0.98 | 7.458 |
| Nrd-3 | 1909.479 | 9.637 | 0.993 | 1336.744 | 0.989 | 7.889 |
| Nrd-3 | 2665.198 | 9.697 | 0.99 | 1421.95 | 0.986 | 7.76 |
| Nrd-3 | 2865.175 | 9.759 | 0.989 | 1399.343 | 0.984 | 7.767 |
| Lrd-1 | 1822.511 | 9.551 | 0.996 | 1040.654 | 0.977 | 6.811 |
| Lrd-1 | 1534.613 | 9.241 | 0.996 | 992.962 | 0.974 | 6.869 |
| Lrd-1 | 1661.451 | 9.444 | 0.996 | 1160.312 | 0.975 | 6.759 |
| Lrd-2 | 1603.413 | 8.977 | 0.98 | 981.1 | 0.958 | 7.045 |
| Lrd-2 | 2724.81 | 9.408 | 0.984 | 1036.552 | 0.956 | 6.992 |
| Lrd-2 | 1505.286 | 8.952 | 0.982 | 1213.888 | 0.962 | 7.021 |
| Lrd-3 | 2100.586 | 8.734 | 0.965 | 1549.277 | 0.984 | 8.097 |
| Lrd-3 | 1723.75 | 8.822 | 0.975 | 1611.523 | 0.984 | 8.065 |
| Lrd-3 | 1660.333 | 8.364 | 0.954 | 1546.056 | 0.983 | 8.067 |
| Mrd-1 | 2512.553 | 9.643 | 0.988 | 969.165 | 0.935 | 6.277 |
| Mrd-1 | 1492.792 | 9.057 | 0.986 | 994.124 | 0.968 | 6.669 |
| Mrd-1 | 1805.279 | 9.257 | 0.986 | 913 | 0.975 | 7.063 |
| Mrd-2 | 1676.853 | 9.159 | 0.989 | 1020.132 | 0.983 | 7.317 |
| Mrd-2 | 2862.96 | 9.599 | 0.989 | 1127.839 | 0.985 | 7.409 |
| Mrd-2 | 2034.772 | 9.353 | 0.986 | 989.551 | 0.984 | 7.242 |
| Mrd-3 | 2805.113 | 10.091 | 0.998 | 797.767 | 0.977 | 6.792 |
| Mrd-3 | 2513.163 | 10.059 | 0.998 | 939.429 | 0.971 | 6.628 |
| Mrd-3 | 1764.697 | 9.658 | 0.996 | 830.25 | 0.976 | 6.773 |
| Srd-1 | 2246.955 | 9.495 | 0.991 | 1342.2 | 0.975 | 7.39 |
| Srd-1 | 2324.862 | 9.562 | 0.991 | 1444.558 | 0.961 | 7.094 |
| Srd-1 | 2361.65 | 9.506 | 0.989 | 1187.811 | 0.972 | 7.251 |
| Srd-2 | 2200.923 | 8.916 | 0.978 | 1154.462 | 0.932 | 6.641 |
| Srd-2 | 2299.233 | 9.261 | 0.985 | 1139.875 | 0.937 | 6.848 |
| Srd-2 | 2154.57 | 8.78 | 0.972 | 1294.51 | 0.939 | 6.777 |
| Srd-3 | 2621.894 | 9.438 | 0.981 | 1130.043 | 0.952 | 6.519 |
| Srd-3 | 1506.073 | 8.932 | 0.981 | 1121.283 | 0.942 | 6.312 |
| Srd-3 | 2409.612 | 9.363 | 0.982 | 1104.03 | 0.946 | 6.388 |

**Table S6**

Coordinate data for PCoA, PCA, and NMDS (Bacterial).

| Group | Sample plot | PCoA | | PCA | | | | | NMDS | |
| --- | --- | --- | --- | --- | --- | --- | --- | --- | --- | --- |
|  |  | PC1 | PC2 | Axis1 | Axis2 | Axis3 | Axis4 | Axis5 | NMDS1 | NMDS2 |
| NRD | Nrd-1 | 0.078 | -0.011 | 10.073 | 29.528 | -28.467 | -5.945 | -14.881 | -0.0877 | -0.0457 |
| NRD | Nrd-1 | 0.077 | -0.024 | 5.216 | 17.952 | -19.223 | -4.540 | -7.944 | -0.0964 | -0.0722 |
| NRD | Nrd-1 | 0.070 | -0.020 | 7.543 | 28.378 | -26.036 | -4.898 | -12.544 | -0.0824 | -0.0532 |
| NRD | Nrd-2 | 0.186 | -0.033 | 40.041 | 45.453 | -25.617 | -3.800 | -30.995 | -0.1797 | -0.0461 |
| NRD | Nrd-2 | 0.179 | -0.056 | 34.221 | 37.503 | -21.209 | -4.708 | -23.861 | -0.2075 | -0.0995 |
| NRD | Nrd-2 | 0.178 | -0.040 | 36.911 | 44.300 | -24.219 | -4.270 | -31.411 | -0.1802 | -0.0564 |
| NRD | Nrd-3 | -0.059 | 0.148 | -10.604 | 33.615 | -15.586 | -2.652 | 3.369 | 0.0875 | 0.1486 |
| NRD | Nrd-3 | -0.041 | 0.110 | -13.909 | 81.345 | -49.736 | -9.218 | 31.408 | 0.0656 | 0.1182 |
| NRD | Nrd-3 | -0.060 | 0.105 | -16.160 | 103.374 | -58.902 | -20.648 | 47.107 | 0.0776 | 0.1022 |
| LRD | Lrd-1 | -0.202 | 0.357 | -30.075 | 23.747 | 0.755 | -21.963 | 20.172 | 0.3520 | 0.2605 |
| LRD | Lrd-1 | -0.195 | 0.374 | -25.254 | 17.723 | 0.986 | -18.636 | 16.458 | 0.3631 | 0.3125 |
| LRD | Lrd-1 | -0.196 | 0.370 | -26.953 | 20.296 | 0.945 | -19.283 | 16.293 | 0.3426 | 0.2802 |
| LRD | Lrd-2 | -0.078 | -0.068 | -12.549 | -7.411 | -1.064 | 6.370 | 3.268 | 0.0557 | -0.0685 |
| LRD | Lrd-2 | -0.066 | -0.033 | -14.693 | -13.934 | -0.996 | 15.190 | 5.405 | 0.0464 | -0.0267 |
| LRD | Lrd-2 | -0.073 | -0.040 | -11.398 | -7.113 | -1.214 | 7.773 | 3.395 | 0.0538 | -0.0406 |
| LRD | Lrd-3 | -0.187 | -0.090 | -30.042 | 4.132 | 12.385 | -20.718 | 6.438 | 0.1458 | -0.1442 |
| LRD | Lrd-3 | -0.195 | -0.094 | -26.728 | 3.017 | 9.125 | -16.226 | 4.974 | 0.1573 | -0.1452 |
| LRD | Lrd-3 | -0.185 | -0.114 | -24.986 | 3.741 | 9.250 | -15.690 | 4.464 | 0.1548 | -0.1884 |
| MRD | Mrd-1 | -0.061 | 0.076 | -8.077 | -2.879 | -8.785 | 16.764 | 13.463 | 0.0490 | 0.0352 |
| MRD | Mrd-1 | -0.064 | 0.086 | -7.956 | -2.233 | -5.652 | 7.346 | 9.600 | 0.0560 | 0.0436 |
| MRD | Mrd-1 | -0.047 | 0.041 | -9.515 | -6.494 | -9.627 | 11.412 | 9.902 | 0.0351 | 0.0308 |
| MRD | Mrd-2 | -0.092 | -0.002 | -15.885 | -13.683 | 1.573 | 35.051 | 14.633 | 0.1382 | 0.0860 |
| MRD | Mrd-2 | -0.092 | -0.007 | -23.039 | -26.643 | 4.529 | 80.115 | 34.138 | 0.1007 | 0.0344 |
| MRD | Mrd-2 | -0.106 | -0.025 | -18.743 | -16.820 | 3.496 | 43.881 | 19.909 | 0.1098 | 0.0199 |
| MRD | Mrd-3 | 0.180 | 0.149 | 15.806 | -36.475 | -34.710 | 88.724 | 35.303 | -0.1755 | 0.2419 |
| MRD | Mrd-3 | 0.177 | 0.155 | 14.044 | -30.125 | -28.313 | 70.076 | 28.158 | -0.1724 | 0.2513 |
| MRD | Mrd-3 | 0.170 | 0.125 | 8.905 | -21.998 | -20.133 | 46.566 | 18.711 | -0.1728 | 0.2232 |
| SRD | Srd-1 | 0.250 | -0.088 | 37.480 | -37.625 | -3.810 | -18.453 | -3.763 | -0.3237 | -0.0655 |
| SRD | Srd-1 | 0.255 | -0.092 | 38.538 | -42.511 | -5.645 | -20.701 | -1.770 | -0.3144 | -0.0551 |
| SRD | Srd-1 | 0.250 | -0.110 | 38.218 | -41.812 | -5.184 | -20.626 | -6.209 | -0.3059 | -0.0787 |
| SRD | Srd-2 | -0.002 | -0.210 | -9.133 | -13.811 | 11.224 | 0.304 | -34.052 | -0.0106 | -0.2192 |
| SRD | Srd-2 | -0.002 | -0.188 | -10.369 | -14.210 | 14.874 | 1.564 | -37.948 | 0.0009 | -0.2195 |
| SRD | Srd-2 | 0.000 | -0.219 | -8.248 | -13.189 | 12.495 | 0.229 | -32.448 | -0.0094 | -0.2040 |
| SRD | Srd-3 | -0.201 | -0.042 | -33.287 | 14.257 | 12.081 | -2.202 | 5.518 | 0.1358 | -0.0419 |
| SRD | Srd-3 | -0.208 | -0.040 | -23.025 | 6.532 | 6.418 | -1.718 | 1.560 | 0.1642 | -0.0362 |
| SRD | Srd-3 | -0.210 | -0.045 | -32.605 | 11.828 | 10.162 | -1.370 | 5.859 | 0.1504 | -0.0420 |

**Table S7**

Coordinate data for PCoA, PCA, and NMDS (Fungal).

| Group | Sample | PCoA | | PCA | | | | | NMDS | |
| --- | --- | --- | --- | --- | --- | --- | --- | --- | --- | --- |
|  |  | PC1 | PC2 | Axis1 | Axis2 | Axis3 | Axis4 | Axis5 | NMDS1 | NMDS2 |
| NRD | Nrd-1 | -0.0761 | -0.2747 | 20.709 | 40.777 | -11.693 | 8.201 | -123.390 | 0.258 | 0.299 |
| NRD | Nrd-1 | -0.0905 | -0.2818 | 18.340 | 32.217 | -10.996 | 5.970 | -88.901 | 0.206 | 0.280 |
| NRD | Nrd-1 | -0.0919 | -0.2675 | 15.511 | 29.124 | -9.615 | 5.702 | -79.692 | 0.216 | 0.303 |
| NRD | Nrd-2 | -0.1571 | -0.2902 | 44.555 | 86.363 | -12.348 | -15.650 | 42.872 | 0.123 | 0.248 |
| NRD | Nrd-2 | -0.1204 | -0.3044 | 53.340 | 109.380 | -17.350 | -17.061 | 63.501 | 0.168 | 0.216 |
| NRD | Nrd-2 | -0.1332 | -0.3057 | 44.111 | 87.021 | -13.995 | -11.954 | 44.155 | 0.160 | 0.233 |
| NRD | Nrd-3 | -0.0541 | -0.1077 | 10.188 | 22.090 | -8.558 | 6.971 | -9.518 | 0.045 | 0.065 |
| NRD | Nrd-3 | -0.0528 | -0.1145 | 10.625 | 21.217 | -8.449 | 7.837 | -12.770 | 0.056 | 0.064 |
| NRD | Nrd-3 | -0.0368 | -0.1190 | 11.692 | 23.818 | -9.356 | 9.021 | -13.455 | 0.076 | 0.057 |
| LRD | Lrd-1 | 0.0569 | 0.1276 | -8.544 | -2.565 | -2.142 | 0.313 | -4.702 | -0.195 | -0.218 |
| LRD | Lrd-1 | 0.0462 | 0.1303 | -8.027 | -2.902 | -2.610 | 0.217 | -4.796 | -0.171 | -0.186 |
| LRD | Lrd-1 | 0.0520 | 0.1198 | -8.790 | -3.081 | -3.308 | 0.627 | -4.751 | -0.139 | -0.174 |
| LRD | Lrd-2 | 0.0376 | 0.1469 | -8.621 | -9.006 | -5.207 | -5.374 | -0.340 | -0.065 | -0.081 |
| LRD | Lrd-2 | 0.0209 | 0.1477 | -8.844 | -9.118 | -5.515 | -5.628 | -0.273 | -0.070 | -0.060 |
| LRD | Lrd-2 | 0.0190 | 0.1690 | -9.307 | -9.693 | -6.197 | -5.198 | -0.831 | -0.078 | -0.054 |
| LRD | Lrd-3 | -0.1862 | 0.1424 | -75.441 | 38.337 | 88.957 | 18.617 | 4.780 | -0.165 | 0.099 |
| LRD | Lrd-3 | -0.2228 | 0.1558 | -69.320 | 33.081 | 79.311 | 15.729 | 4.244 | -0.184 | 0.115 |
| LRD | Lrd-3 | -0.2322 | 0.1316 | -78.718 | 42.352 | 96.542 | 24.618 | 4.539 | -0.192 | 0.143 |
| MRD | Mrd-1 | 0.3099 | 0.0899 | -2.348 | -8.435 | -6.633 | -1.419 | -2.069 | 0.109 | -0.224 |
| MRD | Mrd-1 | 0.2808 | 0.0586 | -2.634 | -8.561 | -7.246 | -0.745 | -1.437 | 0.113 | -0.138 |
| MRD | Mrd-1 | 0.3055 | 0.0886 | -2.759 | -7.186 | -5.608 | -1.359 | -2.920 | 0.058 | -0.116 |
| MRD | Mrd-2 | 0.3033 | 0.1326 | -6.955 | -13.918 | -10.960 | -3.713 | -1.688 | 0.107 | -0.302 |
| MRD | Mrd-2 | 0.2868 | 0.1471 | -6.845 | -14.058 | -12.205 | -3.596 | -0.603 | 0.050 | -0.263 |
| MRD | Mrd-2 | 0.3035 | 0.1361 | -5.956 | -12.789 | -9.927 | -3.273 | -0.594 | 0.089 | -0.295 |
| MRD | Mrd-3 | 0.3711 | 0.1092 | -3.048 | -11.006 | -8.068 | -2.303 | -2.934 | 0.142 | -0.421 |
| MRD | Mrd-3 | 0.3888 | 0.0980 | -3.122 | -10.881 | -8.624 | -2.520 | -3.732 | 0.158 | -0.416 |
| MRD | Mrd-3 | 0.3736 | 0.1020 | -2.924 | -10.309 | -7.809 | -2.441 | -3.918 | 0.158 | -0.405 |
| SRD | Srd-1 | -0.2852 | 0.0501 | -2.619 | -6.963 | -14.523 | -16.627 | 9.185 | -0.163 | 0.334 |
| SRD | Srd-1 | -0.2813 | 0.0345 | -3.323 | -5.613 | -13.977 | -16.023 | 9.795 | -0.156 | 0.363 |
| SRD | Srd-1 | -0.2971 | 0.0467 | -3.613 | -5.612 | -12.137 | -15.123 | 7.811 | -0.161 | 0.342 |
| SRD | Srd-2 | -0.2725 | 0.1072 | -8.373 | -8.017 | -8.351 | -9.638 | 6.359 | -0.183 | 0.257 |
| SRD | Srd-2 | -0.2795 | 0.0944 | -9.289 | -7.548 | -9.649 | -8.769 | 5.713 | -0.154 | 0.263 |
| SRD | Srd-2 | -0.2738 | 0.1102 | -9.376 | -8.467 | -9.429 | -10.592 | 7.670 | -0.192 | 0.268 |
| SRD | Srd-3 | -0.1380 | 0.1600 | -10.263 | -5.013 | -5.248 | -3.378 | -0.979 | -0.340 | -0.047 |
| SRD | Srd-3 | -0.1311 | 0.1534 | -9.211 | -4.254 | -4.474 | -3.324 | -0.428 | -0.360 | -0.059 |
| SRD | Srd-3 | -0.1288 | 0.1663 | -10.305 | -5.072 | -4.838 | -3.778 | -0.519 | -0.346 | -0.063 |

**Table S8**

Plant functional traits and functional diversity indexes of the sample plots.

| Sample plot | dominant species | Functional traits | | | | | | Functional diversity index | | | | |
| --- | --- | --- | --- | --- | --- | --- | --- | --- | --- | --- | --- | --- |
|  |  | H  (m) | LMA  (g·m^-2^) | C  (g·kg^-1^) | N  (g·kg^-1^) | P  (g·kg^-1^) | Si  (g·kg^-1^) | FRic | FEve | FDiv | FDis | Rao |
| Nrd-1 | *Koelreuteria bipinnata* | 15.000 | 0.007 | 397.625 | 12.339 | 0.246 | 3.640 | 2.416 | 0.835 | 0.746 | 1.795 | 3.711 |
| Nrd-1 | *Juglans regia* | 6.500 | 0.007 | 489.718 | 13.048 | 0.268 | 4.584 |  |  |  |  |  |
| Nrd-1 | *Talinum paniculatum* | 0.550 | 0.003 | 1496.241 | 9.652 | 0.321 | 2.546 |  |  |  |  |  |
| Nrd-2 | *Bryophyllum pinnatum* | 0.530 | 0.007 | 396.542 | 7.952 | 0.296 | 2.692 |  |  |  |  |  |
| Nrd-2 | *Tilia tuan* | 8.000 | 0.007 | 400.876 | 17.808 | 0.166 | 3.321 | 18.687 | 0.698 | 0.679 | 2.016 | 5.297 |
| Nrd-2 | *Cipadessa baccifera* | 4.500 | 0.003 | 442.047 | 16.520 | 0.247 | 3.069 |  |  |  |  |  |
| Nrd-3 | *Triadica rotundifolia* | 12.000 | 0.006 | 472.383 | 12.516 | 0.127 | 3.753 |  |  |  |  |  |
| Nrd-3 | *Bambusa emeiensis* | 18.000 | 0.005 | 472.383 | 15.400 | 0.174 | 15.757 | 10.705 | 0.644 | 0.649 | 2.225 | 6.591 |
| Nrd-3 | *Celtis biondii* | 3.700 | 0.011 | 409.543 | 8.400 | 0.108 | 10.373 |  |  |  |  |  |
| Lrd-1 | *Solanum erianthum* | 3.800 | 0.005 | 374.873 | 19.264 | 0.232 | 2.944 |  |  |  |  |  |
| Lrd-1 | *Vitex negundo* var. cannabifolia | 1.800 | 0.006 | 520.736 | 15.288 | 0.230 | 2.818 | 3.740 | 0.832 | 0.769 | 1.940 | 4.142 |
| Lrd-1 | *Alangium chinense* | 3.100 | 0.007 | 484.032 | 9.800 | 0.228 | 3.139 |  |  |  |  |  |
| Lrd-2 | *Cipadessa baccifera* | 2.000 | 0.005 | 572.350 | 11.816 | 0.268 | 2.660 |  |  |  |  |  |
| Lrd-2 | *Morus mongolica* | 2.800 | 0.009 | 488.620 | 11.424 | 0.160 | 11.875 | 1.113 | 0.775 | 0.766 | 1.791 | 3.613 |
| Lrd-2 | *Tilia miqueliana* | 4.000 | 0.008 | 478.297 | 12.656 | 0.291 | 3.433 |  |  |  |  |  |
| Lrd-3 | *Paraboea rufescens* | 0.110 | 0.010 | 505.825 | 6.384 | 0.148 | 4.454 |  |  |  |  |  |
| Lrd-3 | *Solanum erianthum* | 2.500 | 0.005 | 486.326 | 18.368 | 0.247 | 2.694 | 0.392 | 0.787 | 0.727 | 1.676 | 3.145 |
| Lrd-3 | *Mallotus philippensis* | 2.750 | 0.008 | 446.755 | 13.160 | 0.188 | 6.543 |  |  |  |  |  |
| Mrd-1 | *Arthraxon lanceolatus* | 0.270 | 0.003 | 354.829 | 10.584 | 0.364 | 13.428 |  |  |  |  |  |
| Mrd-1 | *Celtis bungeana* | 3.500 | 0.008 | 407.376 | 6.468 | 0.093 | 11.137 | 0.274 | 0.809 | 0.750 | 1.627 | 2.995 |
| Mrd-1 | *Morus alba* | 3.500 | 0.008 | 379.207 | 10.808 | 0.168 | 7.552 |  |  |  |  |  |
| Mrd-2 | *Ailanthus altissima* | 7.000 | 0.006 | 378.123 | 10.808 | 0.159 | 3.445 |  |  |  |  |  |
| Mrd-2 | *Alchornea trewioides* | 1.620 | 0.007 | 398.709 | 9.016 | 0.161 | 2.822 |  |  |  |  |  |
| Mrd-2 | *Vitex negundo* | 1.500 | 0.008 | 486.468 | 10.080 | 0.163 | 2.696 | 3.066 | 0.828 | 0.746 | 1.877 | 3.973 |
| Mrd-3 | *Mallotus philippensis* | 5.000 | 0.009 | 459.382 | 7.560 | 0.128 | 11.142 |  |  |  |  |  |
| Mrd-3 | *Ageratina adenophora* | 0.720 | 0.006 | 497.302 | 9.828 | 0.184 | 2.656 | 0.656 | 0.765 | 0.774 | 1.957 | 4.189 |
| Srd-1 | *Praxelis clematidea* | 0.570 | 0.002 | 424.711 | 15.176 | 0.389 | 4.885 | 0.019 | 0.866 | 0.741 | 2.032 | 4.715 |
| Srd-1 | *Bauhinia championii* | 2.000 | 0.009 | 411.710 | 10.304 | 0.120 | 3.959 |  |  |  |  |  |
| Srd-1 | *Ipomoea nil* | 1.000 | 0.002 | 343.453 | 11.592 | 0.417 | 3.092 |  |  |  |  |  |
| Srd-2 | *Bidens alba* | 0.560 | 0.004 | 385.707 | 14.336 | 0.302 | 3.478 | 0.039 | 0.893 | 0.798 | 2.238 | 5.326 |
| Srd-2 | *Cladrastis platycarpa* | 2.500 | 0.008 | 453.964 | 12.992 | 0.181 | 3.219 |  |  |  |  |  |
| Srd-3 | *Erigeron annuus* | 0.500 | 0.003 | 408.460 | 13.048 | 0.514 | 7.428 | 0.010 | 0.792 | 0.723 | 1.698 | 3.247 |
